# Readily Available Chiral Benzimidazoles-Derived Guanidines as Organocatalysts in the Asymmetric α-Amination of 1,3-Dicarbonyl Compounds

**DOI:** 10.3390/molecules22081333

**Published:** 2017-08-11

**Authors:** Llorenç Benavent, Francesco Puccetti, Alejandro Baeza, Melania Gómez-Martínez

**Affiliations:** Departamento de Química Orgánica and Instituto de Síntesis Orgánica (ISO), Facultad de Ciencias, Universidad de Alicante, Apdo. 99, E-03080 Alicante, Spain; l.benavent@ua.es (L.B.); francesco.puccetti@stud.unifi.it (F.P.); melania.gomez@ua.es (M.G.-M.)

**Keywords:** organocatalysis, electrophilic amination, guanidines, asymmetric catalysis, dicarbonyl compounds

## Abstract

The synthesis and the evaluation as organocatalysts of new chiral guanidines derived from benzimidazoles in the enantioselective α-amination of 1,3-dicarbonyl compounds using di-*t*-butylazodicarboxylate as aminating agent is herein disclosed. The catalysts are readily synthesized through the reaction of 2-chlorobezimidazole and a chiral amine in moderate-to-good yields. Among all of them, those derived from (*R*)-1-phenylethan-1-amine (**1**) and (*S*)-1-(2-naphthyl)ethan-1-amine (**3**) turned out to be the most efficient for such asymmetric transformation, rendering good-to-high yields and moderate-to-good enantioselectivities for the amination products.

## 1. Introduction

The synthesis of chiral molecules bearing a nitrogen-containing quaternary stereocenter adjacent to a carboxylic acid derivative in a direct and catalytic manner is still considered a challenging transformation [1,2,3]. Among the different strategies developed to accomplish this, the electrophilic amination of 1,3-dicarbonyl compounds [4,5,6,7] has emerged as an attractive methodology. Since the pioneering work developed by the Jørgensen group, who published a metal- [8] and an organocatalyzed [9] amination of β-keto esters and β-keto nitriles with azodicarboxylates, the work done in this field has grown considerably [10,11,12,13,14,15]. In the last years, the work developed in the field of organocatalysis for this transformation has been quite prolific [16,17,18,19,20], especially thanks to the irruption of the hydrogen-bond organocatalysis, which has resulted in the publication of several works employing this strategy [21,22,23,24,25,26,27,28,29].

**Figure 1:**
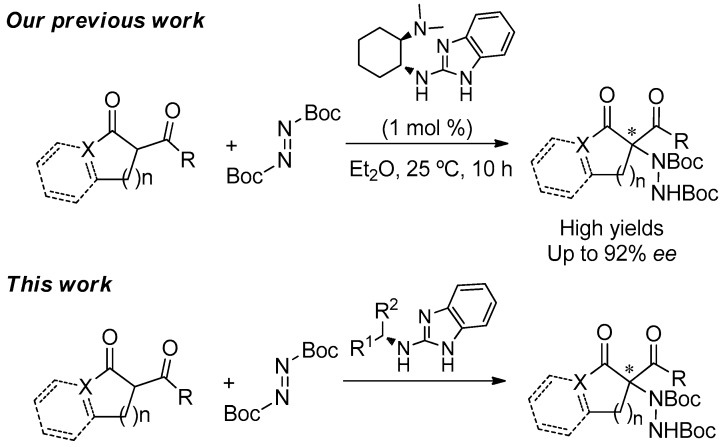
Benzimidazoles-based guanidine organocatalyst in the asymmetric electrophilic aminations of dicarbonyl compounds.

In the last decade, our research group has been involved in the synthesis and application of benzimidazole-derivatives as bifunctional organocatalysts able to activate different 1,3-dicarbonyl compounds through hydrogen-bond interactions in different asymmetric organocatalyzed transformations [30,31,32,33,34]. Thus, although such benzimidazoles have recently demonstrated to be efficient catalysts for the electrophilic amination of dicarbonyl compounds [35], we decided to further extend the investigations in this difficult transformation by synthesizing new and readily available guanidines derived from benzimidazoles and evaluating their catalytic performance (Figure 1).

## 2. Results and Discussion

**Figure 2:**
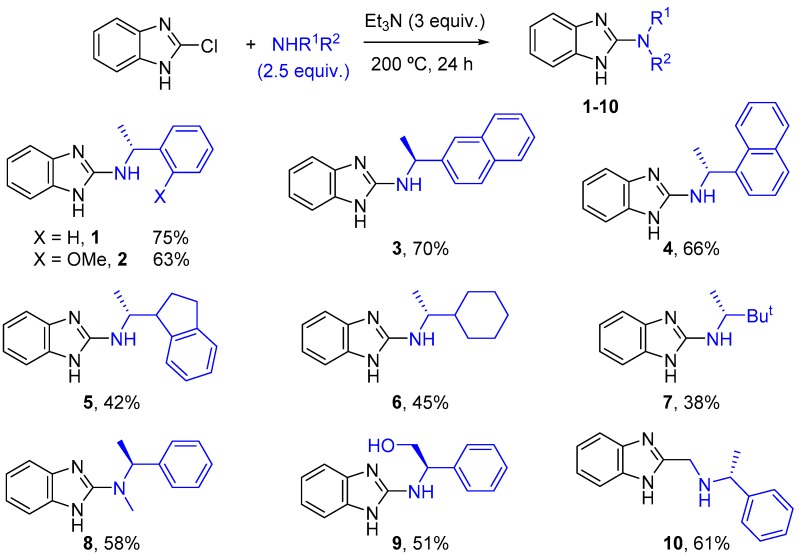
Synthesis of chiral organocatalysts.

Firstly, a series of new different guanidine organocatalysts were synthesized following a straightforward methodology developed in our research group [30,31,32,33,34,35]. Thus, 2-chlorobenzimidazole was reacted with the corresponding chiral amine in the presence of Et_3_N (3 equiv.) at 200 °C (pressure tube) for 24 h (Figure 2). Under these conditions, the corresponding products (**1**–**10**) were obtained in moderate-to-good yields. As expected, lower yields were achieved when bulky aliphatic amines were employed (**5**–**7**). For the synthesis of benzimidazole **10**, the corresponding 2-chloromehtylbenzimidazole was used instead.

Once the guanidine derivatives were synthesized, their performance as organocatalysts in the asymmetric electrophilic amination of 1,3-dicarbonyl compounds was evaluated. In the search for optimal conditions and catalyst, and based on our previous experience in this transformation [35], the reaction between ethyl 2-oxocyclopenthanecarboxylate and di-*t*-butylazodicarboxylate in toluene at 25 °C was chosen as a model reaction using 10 mol % of guanidine-derivative loading (Table 1). Thus, catalysts bearing an aromatic ring in the amine moiety (**1**–**4**) produced amination product **13a** in high conversions and good-to-high enantioselectivities (Table 1, entries **1**–**4**), whereas those bearing an aliphatic moiety (**5**–**7**) gave rise to **13a** in low or moderate conversions and *ee*’s (Table 1, entries **5**–**7**). The influence of the hydrogens in the guanidine moiety was evaluated with catalyst **8** lacking one of them and bearing a tertiary amine part. As can be observed, although the results were quite good, the presence of an additional hydrogen—which could play an important role in the substrate activation—seems to be crucial in order to achieve high conversions and enantioselectivities (Table 1, entry **8**). By the contrary, the introduction of an extra activation point by using an aminoalcohol moiety as in catalyst **9** turned out to be detrimental for the reaction results (Table 1, entry **9**). Catalyst **10** lacks conjugation between nitrogens in the guanidine moiety, and gave rise to both low conversion and enantioselection for compound **13a**.

According to the catalyst screening results, we decided to further optimize the reaction conditions with guanidine **1**, since it performed the best in terms of both conversion and enantioselectivity for compound **13a**. In this regard, the influence of the solvent, temperature, and catalyst loading was assessed (Table 2). As can be observed, although good conversions were reached, none of the solvents tested produced an amelioration in the enantioselectivity (Table 2, entries **1**–**4**). Then, once toluene was selected as the optimal solvent, lowering the reaction temperature was the next parameter assayed. Thus, it was found that while *ee* barely changed at 0 °C, the conversion dropped down to 87% (Table 2, entry **5**). Finally, a decrease in the catalyst loading (5 mol %) was evaluated not only with catalyst **1** but also with **3**, which also gave satisfactory results in the amination reaction. However, a slight drop in the enantioselectivity of the process was observed (Table 2, entries **6** and **7**).

The influence of the substituent in the diazocompounds was also tested. In this regard, diethyl- and dibenzylazodicarboxylate were screened, but both of them gave rise to the corresponding amination products in lower yields and *ee*’s (see Supporting Information for details).

**Figure 3:**
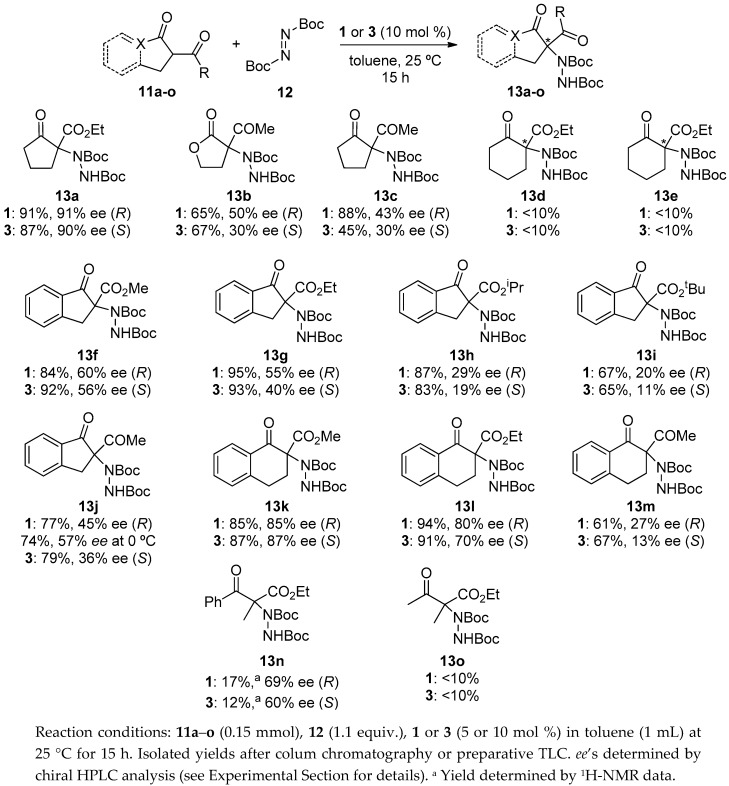
Scope of the reaction.

After having screened several parameters, the optimal reaction conditions chosen were the use of benzimidazole **1** (10 mol %) as catalyst, toluene as solvent, and 25 °C as temperature. In addition, since catalyst **3** performed similarly to **1**, it was also selected for testing the substrate scope of the reaction (Figure 3). Thus, cyclic five-membered 1,3-dicarbonyl compounds were firstly evaluated. As mentioned, good results were achieved for **13a**. However, when substrates **11b** and **11c** were tested, the corresponding products **13b** and **13c** were only isolated in moderate-to-good yields and enantioselectivities [36]. Unfortunately, six-membered dicarbonyl compounds turned out to be unreactive under these conditions [36]. Next, indanone-derived β-keto esters were assayed. As can be observed in Figure 2, increasing the steric bulk at the alkyl substituent of the ester moiety resulted in lower enantioselectivities and yields, obtaining the best enantioselection for methyl ester derivative **13f** (60% *ee*). As somehow expected, the more-reactive 2-acetyl-1-indanone gave rise to the amination product **13j** in moderate enantioselectivity, although in unexpectedly slightly lower yield than its keto ester analogue. Next, tetralone derivatives were taken into account. Contrary to the behavior observed with their non-benzocondensed analogues, very high enantioselectivities were achieved along with good yields. Again, **13k**, bearing a methyl ester moiety, was superior in terms of *ee* in front of **13l** (85% and 80% *ee*, respectively with catalyst **1**). Lower yields and enantioselectivities were obtained for product **13m** when the more reactive diketone was employed, being the drop even more accused in this case. Finally, open-chained 1.3-keto esters—which normally fail with most of the published catalysts [35]—were assayed. To our surprise, the use of ethyl benzoyl acetate gave rise to the corresponding amination product in good *ee’*s, although with poor yields. Despite this, encouraged by the good enantioselectivities reached for **13n** we decided to test another acyclic ketoester: methyl 3-oxo-2-methylbutanoate. Disappointingly, the reaction failed completely with both catalysts. It is important to mention that with the aim of obtaining better results a decrease in reaction temperature was assayed in the majority of the cases where compounds **13** were achieved with moderate *ee*’s and good yields. However, with the exception of compound **13j**, where the *ee* increased significantly, no substantial enhancement of optical purity was observed and, in addition, a drop in the yield was observed in most cases.

**Figure 4:**
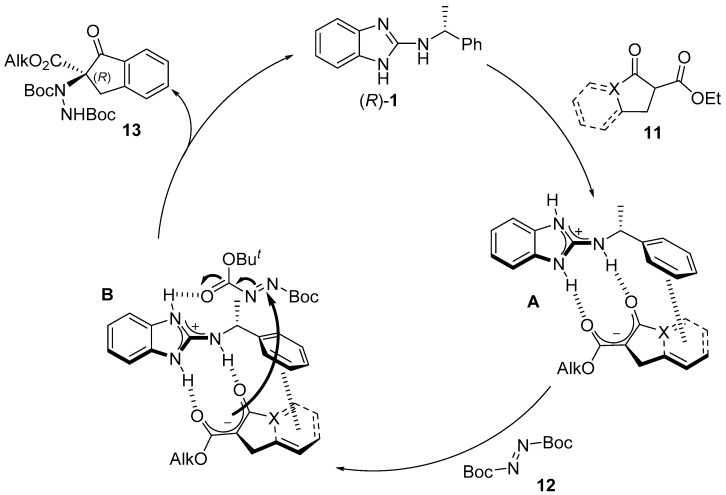
Proposed reaction mechanism.

In order to gain a deeper knowledge about the reaction mechanism, nonlinear effect experiments were conducted to determine whether one or more catalyst molecules are involved in the catalytic cycle [37]. According to the observed results, it was determined that such a nonlinear effect did not occur in the studied reaction, pointing towards the involvement of a single catalyst molecule operating in the catalytic cycle (see Supporting Information for details about the experiments). Thus, a hypothetical model for the catalytic cycle is depicted in Figure 4, which is based on previous computational and experimental studies carried out in our research group employing benzimidazole-derived catalysts in asymmetric conjugate additions. Thus, guanidine benzimidazole-derived (*R*)-**1** could act as a bifunctional organocatalyst, acting initially as a base. Then, the 1,3-dicarbonyl compound enolate formed could be coordinated with the organocatalyst through hydrogen bonding, as depicted in intermediate **A**. A possible π–π stacking interaction would explain the good results observed in the benzocondensed β-keto esters, and could not be ruled out. Afterwards, the protonated guanidine group can activate the azodicarboxylate and hence facilitate the enantioselective attack of the enolate (intermediate **B**), releasing the corresponding amination product and regenerating (*R*)-**1**. It is important to remark that (*R*)-configured amination product was obtained when (*R*)-**1** is employed. This assumption was taken by comparison of experimental evidence (chiral HPLC charts and optical rotation) with those previously reported in the literature for the same products. As expected, when (*S*)-**3** was the catalyst employed, the opposite absolute configuration was observed in the amination product.

## 3. Materials and Methods

All reagents were purchased from commercial sources and used without further purification. Substrates that were not commercially available were synthesized according to known literature procedures [19,22,35]. IRs were recorded on a JASCO FT-IR 4100 LE (Pike Miracle ATR) (Jasco Analítica Spain, Madrid, Spain), and only the structurally most relevant peaks are listed. NMR spectra were performed on a Bruker AC-300 or Bruker Avance-400 400 (Bruker Corporation, Billerica, MA, USA) using CDCl_3_ as solvent and tetramethylsilane (TMS) as internal standard unless otherwise stated. Low-resolution electron impact (EI) mass spectra were obtained at 70 eV on Agilent GC/MS-5973N apparatus equipped with a HP-5MS column (Agilent technologies, 30 m × 0.25 mm). Optical rotations were measured on a JASCO P-1030 Polarimeter (Jasco Analítica Spain, Madrid, Spain) with a 5 cm cell (c given in g/100 mL). Enantioselectivities were determined by HPLC analysis (Agilent 1100 Series HPLC) equipped with a G1315B diode array detector and a Quat Pump G1311A (Agilent Technologies, Santa Clara, CA, USA) equipped with the corresponding Daicel chiral column, and the retention time of the major enantiomer is highlighted in bold. Analytical thin layer chromatography (TLC) was performed on Merck silica gel plates and the spots visualized with UV light at 254 nm (Merck Millipore, Billerica, MA, USA). Flash chromatography employed Merck silica gel 60 (0.040–0.063 mm) (Merck Millipore).

### 3.1. General Procedure for the Synthesis of Benzimidazole-Based Organocatalysts ***1**–**10***

In a sealed pressure tube, a mixture of 2-chloro-1*H*-benzo[*d*]imidazole (0.5 mmol), the corresponding chiral amine **1** (2.5 equiv.), and Et_3_N (3 equiv.) was heated at 195–200 °C during 24 h. The reaction mixture was then allowed to reach room temperature. After the addition of H_2_O (5 mL), the mixture was extracted with CH_2_Cl_2_ (3 × 5 mL). The combined organic phases were dried over MgSO_4_ and evaporated under reduced pressure to give the corresponding crude products, which were purified by flash chromatography (Hexane/EtOAc/MeOH). For the synthesis of benzimidazole **10**, the same procedure was followed using 2-chloromethylbenzimidazole (0.5 mmol). The data shown below correspond to the pure compounds:

*(R)-N-(1-Phenylethyl)-1H-benzo[d]imidazol-2-amine* (**1**): White powder; [α]_D_^26^ = +78.0 (*c* = 0.75, CHCl_3_); IR (ATR) ν_max_: 3412, 3059, 1565, 1463, 1271, 1133, 1012 cm^−1^; ^1^H-NMR (300 MHz, CDCl_3_) δ_H_ = 1.45 (d, *J* = 6.8 Hz, 3H), 5.00 (q, *J* = 6.8 Hz, 1H), 6.14 (s, 1H), 7.05 (dd, *J* = 5.8, 3.2 Hz, 2H), 7.14–7.30 (m, 7H) ppm; ^13^C-NMR (75 MHz, CDCl_3_) δ_C_ = 24.0, 52.9, 112.3, 120.6, 125.7, 127.3, 128.7, 144.0, 155.1 ppm; MS (EI) *m*/*z* 237 (M^+^, 53%), 138 (100), 105 (65), 77 (17).

*(R)-N-(1-(2-Methoxyphenyl)ethyl)-1H-benzo[d]imidazol-2-amine* (**2**): White powder; [α]_D_^28^ = +67.6 (*c* = 1.01, CHCl_3_); IR (ATR) ν_max_: 3395, 3054, 2963, 1576, 1463, 1258, 1131 cm^−1^; ^1^H-NMR (300 MHz, CDCl_3_) δ_H_ = 1.44 (d, *J* = 6.7 Hz, 3H), 3.35 (s, 3H), 5.34 (m, 1H), 6.02–6.42 (s, 1H), 6.63 (d, *J* = 8.2 Hz, 1H), 6.81 (t, *J* = 7.4 Hz, 1H), 6.96 (m, 2H), 7.14 (m, 3H), 7.25 (m, 1H), 11.05–9.14 (br s, 1H) ppm; ^13^C-NMR (75 MHz, CDCl_3_): δ_C_ = 22.3, 49.0, 54.8, 110.7, 112.1, 120.2, 120.8, 126.5,128.3, 131.6, 155.2, 156.5 ppm; MS (EI) *m*/*z* 267 (M^+^, 25%), 236 (57), 135 (100), 105 (25), 77 (13).

*(S)-N-(1-(Naphthalen-2-yl)ethyl)-1H-benzo[d]imidazol-2-amine* (**3**): Yellowish powder; [α]_D_^25^ = −105.5 (*c* = 1.00, CHCl_3_); IR (ATR) ν_max_: 3392, 3053, 1600, 1571, 1462, 1268, 1130 cm^−1^; ^1^H-NMR (300 MHz, CDCl_3_) δ_H_ = 1.51 (d, *J* = 6.7 Hz, 3H), 4.95 (d, *J* = 6.6 Hz, 1H), 6.03 (s, 1H), 6.96 (dd, *J* = 5.7, 3.2 Hz, 2H), 7.16 (dd, *J* = 5.7, 3.0 Hz, 2H), 7.34–7.47 (m, 3H), 7.60–7.79 (m, 4H), ppm; ^13^C-NMR (75 MHz, CDCl_3_) δ_C_ = 24.0 53.3, 112.3, 120.6, 124.0, 124.1, 125.9, 126.3, 127.6, 127.8, 128.8, 132.8, 133.3, 141.1, 154.6, ppm.

*(R)-N-(1-(Naphthalen-1-yl)ethyl)-1H-benzo[d]imidazol-2-amine* (**4**): Yellowish powder; [α]_D_^23^ = −95.5 (*c* = 0.99, CHCl_3_); IR (ATR) ν_max_: 3405, 3050, 1572, 1462, 1268 cm^−1^;^1^H-NMR (300 MHz, CDCl_3_) δ_H_ = 1.42 (d, *J* = 6.7 Hz, 3H), 5.56 (q, *J* = 6.3 Hz, 1H), 6.09 (s, 1H), 6.86–6.93 (m, 2H), 7.03 (s, 2H), 7.15–7.31 (m, 3H), 7.41 (d, *J* = 7.0 Hz, 1H), 7.66 (m, 1H), 7.85 (d, *J* = 8.5 Hz, 1H) ppm. ^13^C-NMR (75 MHz, CDCl_3_) δ_C_ = 22.6, 49.1, 120.3, 122.0, 122.8, 125.4, 125.6, 126.1, 128.0, 128.8, 130.6, 133.9, 139.0, 154.7 ppm.

*(R)-N-(2,3-Dihydro-1H-inden-1-yl)-1H-benzo[d]imidazol-2-amine* (**5**): Light brown powder; [α]_D_^26^ = +18.2 (*c* = 0.75, CHCl_3_); IR (ATR) ν_max_: 3043, 2936, 1578, 1461, 1270, 1054 cm^−1^; ^1^H-NMR (300 MHz, CDCl_3_) δ_H_ = 1.83–1.98 (m, 1H), 2.59–2.72 (m, 1H), 2.78–2.91 (m, 1H), 2.92–3.04 (m, 1H), 5.07–5.31 (br s, 1H), 5.39 (t, *J* = 7.1 Hz, 3H), 7.02–7.09 (m, 2H), 7.12–7.20 (m, 1H), 7.22–7.26 (m, 2H), 7.26–7.37 (m, 3H) ppm; ^13^C-NMR (75 MHz, CDCl_3_) δ_C_ = 30.0, 34.3, 58.5, 112.1, 120.9, 123.9, 124.9, 126.7, 128.1, 142.8, 143.1, 154.3 ppm; MS (EI) *m*/*z* 249 (M^+^, 27%), 133 (100), 115 (44), 91 (10).

*(R)-N-(1-Cyclohexylethyl)-1H-benzo[d]imidazol-2-amine* (**6**): White powder; [α]_D_^26^ = +2.7 (*c* = 0.98, CHCl_3_); IR (ATR) ν_max_: 3390, 2924, 2851, 1630, 1577, 1463, 1262, 1156 cm^−1^; ^1^H-NMR (300 MHz, CDCl_3_) δ_H_ = 0.77–1.09 (m, 5H), 1.12 (d, *J* = 6.6 Hz, 3H), 1.29–1.40 (m, 1H), 1.50–1.66 (m, 5H), 3.81 (m, 1H), 5.38 (s, 1H), 7.01 (dd, *J* = 5.8, 3.2 Hz, 2H), 7.25 (dd, *J* = 6.1, 2.9 Hz, 2H) ppm; ^13^C-NMR (75 MHz, CDCl_3_) δ_C_ = 18.3, 26.1, 26.3, 28.7, 29.0, 43.3, 53.6, 111.9, 120.4, 129.8, 137.8, 155.8 ppm; MS (EI) *m*/*z* 243 (M^+^, 24%), 200 (8), 160 (59), 133 (100), 105 (9).

*(R)-N-(3,3-Dimethylbutan-2-yl)-1H-benzo[d]imidazol-2-amine* (**7**): White powder; [α]_D_^28^ = −69.6 (*c* = 0.97, CHCl_3_); IR (ATR) ν_max_: 3054, 2931, 1628, 1572, 1461, 1239, 1027 cm^−1^; ^1^H-NMR (300 MHz, CDCl_3_) δ_H_ = 0.79 (s, 9H) 1.08 (t, *J* = 11.8 Hz, 3H),3.80 (s, 1H), 5.31 (s, 1H), 7.01 (dt, *J* = 10.2, 5.1 Hz, 2H), 7.25 (td, *J* = 6.1, 2.6 Hz, 2H) ppm; ^13^C-NMR (75 MHz, CDCl_3_) δ_C_ = 16.7 26.1, 34.4, 57.4, 111.9, 120.4, 156.4 ppm; MS (EI) *m*/*z* 217 (M^+^, 22%), 223 (100), 133 (17), 105 (4).

*(S)-N-Methyl-N-(1-phenylethyl)-1H-benzo[d]imidazol-2-amine* (**8**): White powder; [α]_D_^27^ = −177.6 (*c* = 1.00, CHCl_3_); IR (ATR) ν_max_: 2975, 1559, 1506, 1418, 1264, 1030, 905 cm^−1^; ^1^H-NMR (300 MHz, CDCl_3_) δ_H_ = 1.51 (d, *J* = 6.9 Hz, 3H), 2.79 (s, 3H), 5.64 (q, *J* = 7.0 Hz, 1H), 7.11 (m, 9H) ppm; ^13^C-NMR (75 MHz, CDCl_3_) δ_C_ = 16.2, 30.6, 55.7, 112.2, 120.4, 126.9, 127.4, 128.6, 140.7, 156.3 ppm; MS (EI) *m*/*z* 251 (M^+^, 86%), 236 (48), 147 (100), 118 (44), 105 (85), 91 (11), 77 (23).

*(R)-2-[(1H-Benzo[d]imidazol-2-yl)amino]-2-phenylethanol* (**9**): White powder; [α]_D_^29^ = −83.0 (*c* = 0.71, MeOH); ^1^H-NMR (300 MHz, CD_3_COD) δ_H_ = 3.80 (dd, *J* = 11.3, 6.9 Hz, 1H), 3.88 (dd, *J* = 11.3, 4.8 Hz, 1H), 4.08 (q, 1H), 6.90–6.97 (m, 2H), 7.13–7.25 (m, 3H), 7.27–7.37 (m, 4H), 7.41–7.47 (m, 2H) ppm; ^13^C-NMR (75 MHz, CD_3_COD) δ_C_ = 24.0, 52.8, 112.2, 120.5, 125.6, 127.3, 128.7, 143.9, 155.0.

*(R)-N-((1H-Benzo[d]imidazol-2-yl)methyl)-1-phenylethanamine* (**10**): White powder; [α]_D_^28^ = −22.6 (*c* = 1.00, CHCl_3_); IR (ATR) ν_max_: 3052, 1424, 1264 cm^−1^; ^1^H-NMR (300 MHz, CDCl_3_) δ_H_ = 1.37 (d, *J* = 6.6 Hz, 3H) 3.80 (q, 1H), 3.90 (2H), 5.35–6.00 (s, 1H), 7.17–7.23 (m, 3H), 7.24–7.29 (m, 3H), 7.31–7.36 (m, 1H), 7.49–7.57 (m, 2H) ppm; ^13^C-NMR (75 MHz, CDCl_3_): δ_C_ = 23.9,45.3, 58.1, 76.6, 77.1, 77.5, 114.9, 122.4, 126.6, 127.3, 128.6, 138.3, 144.2, 153.9.

### 3.2. General Procedure for the Asymmetric Amination of 1,3-Dicarbonyl Compounds

In an open-air tube at room temperature (25 °C) the corresponding 1,3-dicarbonyl compound (0.1 mmol) was added to a solution of organocatalyst (0.01 mmol, 10 mol %) in toluene (1 mL). After 5 min, di-*tert*-butylazodicarboxilate (0.11 mmol, 1.1 equiv.) was added in one portion and the reaction was then allowed to react for 15 h. After this time, water (5 mL) and ethyl acetate were added, and then the aqueous layer was re-extracted twice (2 × 10 mL). The combined organic phases were dried (MgSO_4_) and evaporated under reduced pressure. Finally, the reaction crude was purified by column chromatography on silica gel or preparative TLC using hexane/ethyl acetate mixtures as eluent. The analytical data shown below correspond to those enantioenriched products (≥20% *ee*) as representative compounds. All the compounds are described in the literature. Therefore, only ^1^H-NMR, MS (EI) and enantiomeric excess determination conditions are listed.

*(R)-Di-tert-butyl 1-[1-(ethoxycarbonyl)-2-oxocyclopentyl]hydrazine-1,2-dicarboxylate* (**13a**) [35]: Colorless oil; [α]_D_^28^ = −6.10 (*c* = 0.49, CHCl_3_, 91% *ee*); ^1^H-NMR (300 MHz, CDCl_3_) δ_H_ = 1.28 (t, *J* = 7.1 Hz, 3H), 1.59–1.36 (m, 18H), 2.98–1.75 (m, 6H), 4.24 (m, 2H), 6.53 (br s, 1H) ppm; chiral HPLC analysis: Chiralcel IA column, Hexane/EtOH 96:04, flow rate = 0.7 mL/min, λ = 210 nm, retention times: **9.9** and 11.0 min.

*(R)-Di-tert-butyl 1-(3-acetyl-2-oxotetrahydrofuran-3-yl)hydrazine-1,2-dicarboxylate* (**13b**) [35]: Colorless oil; [α]_D_^28^ = +2.64 (*c* = 1.0, CHCl_3_, 50% *ee*); ^1^H-NMR (300 MHz, CDCl_3_) δ_H_ = 1.47 (d, *J* = 1.9 Hz, 18H), 1.62 (s, 3H), 2.28–2.44 (m, 2H), 4.38 (d, *J* = 6.6 Hz, 2H), 6.79 (br s, 1H) ppm; chiral HPLC analysis: Chiralcel IA column, Hexane/EtOH 98:02, flow rate = 1 mL/min, λ = 210 nm, retention times: **21.0** and 24.2 min.

*(R)-Di-tert-butyl 1-(1-acetyl-2-oxocyclopentyl)hydrazine-1,2-dicarboxylate* (**13c**) [35]: Colorless oil; [α]_D_^28^ = +16.99 (*c* = 1.1, CHCl_3_, 43% *ee*); ^1^H-NMR (300 MHz, CDCl_3_) δ_H_ = 1.45 (d, *J* = 7.2 Hz, 18H), 1.56–2.09 (m, 3H), 2.51–2.18 (m, 5H), 2.84–2.51 (m, 1H), 6.48 (br s, 1H) ppm; chiral HPLC analysis: Chiralcel AD-H column, Hexane/EtOH 98:02, flow rate = 1 mL/min, λ = 210 nm, retention times: **12.2** and 17.3 min.

*(R)-Di-tert-butyl 1-[2-(methoxycarbonyl)-1-oxo-2,3-dihydro-1H-inden-2-yl]hydrazine-1,2-dicarboxylate* (**13f**) [24]: Slightly yellow oil; [α]_D_^28^ = −57.19 (*c* = 1.0, CHCl_3_, 60% *ee*); ^1^H-NMR (300 MHz, CDCl_3_) δ_H_ = 0.78–1.89 (m, 18H), 3.45–4.39 (m, 5H), 6.34–6.84 (m, 1H), 7.36 (t, *J* = 7.4 Hz, 1H), 7.49 (d, *J* = 7.2 Hz, 1H), 7.63 (t, *J* = 7.2 Hz, 1H), 7.77 (d, *J* = 7.6 Hz, 1H) ppm; chiral HPLC analysis: Chiralcel IA column, Hexane/iPrOH 90:10, flow rate = 1 mL/min, λ = 240 nm, retention times: **16.1** and 22.1 min.

*(R)-Di-tert-butyl 1-[2-(ethoxycarbonyl)-1-oxo-2,3-dihydro-1H-inden-2-yl]hydrazine-1,2-dicarboxylate* (**13g**) [35]: Colorless sticky oil; [α]_D_^28^ = −42.40 (*c* = 1.4, CHCl_3_, 55% *ee*); ^1^H-NMR (300 MHz, CDCl_3_) δ_H_ *=* 1.14–1.55 (m, 21H), 3.82 (d, *J* = 16.6 Hz, 1H), 3.96–4.32 (m, 3H), 6.31–6.83 (bs, 1H), 7.36 (t, *J* = 7.3 Hz, 1H), 7.49 (d, *J* = 6.9 Hz, 1H), 7.62 (t, *J* = 7.2 Hz, 1H), 7.76 (d, *J* = 9.4 Hz, 1H) ppm; chiral HPLC analysis: Chiralcel IA column, Hexane/iPrOH 90:10, flow rate = 1 mL/min, λ = 240 nm, retention times: **12.5** and 14.4 min.

*(R)-Di-tert-butyl 1-(2-(isopropoxycarbonyl)-1-oxo-2,3-dihydro-1H-inden-2-yl)hydrazine-1,2-dicarboxylate* (**13h**) [29]: Colorless oil; [α]_D_^28^ = −6.46, (*c* = 1.4, CHCl_3_ 29% *ee*); ^1^H-NMR (300 MHz, CDCl_3_) δ_H_ = 1.12–1.52 (m, 24H), 3.71 (dd, *J* = 69.4, 16.6 Hz, 1H), 4.16 (m, 1H), 5.03 (dt, *J* = 12.4, 6.1 Hz, 1H), 6.32–6.83 (m, 1H), 7.35 (t, *J* = 7.2 Hz, 1H), 7.49 (d, *J* = 7.6 Hz, 1H), 7.61 (t, *J* = 7.2 Hz, 1H), 7.74 (d, *J* = 14.7 Hz, 1H) ppm; chiral HPLC analysis: Chiralcel AD-H column, Hexane/EtOH 96:04, flow rate = 1 mL/min, λ = 254 nm, retention times: **11.2** and 28.5 min.

*(R)-Di-tert-butyl 1-(2-(tert-butoxycarbonyl)-1-oxo-2,3-dihydro-1H-inden-2-yl)hydrazine-1,2-dicarboxylate* (**13i**) [29]: Colorless oil; [α]_D_^28^ = −3.20, (*c* = 1.0, CHCl_3_, 21% *ee*); ^1^H-NMR (300 MHz, CDCl_3_) δ_H_ *=* 1.55–1.21 (m, 27H), 3.22 (d, *J* = 17.2 Hz, 1H), 3.65 (d, *J* = 17.2 Hz, 1H), 6.62 (br s, 1H), 7.36 (d, *J* = 7.4 Hz, 1H), 7.48 (d, *J* = 7.7 Hz, 1H), 7.59–7.69 (m, 1H), 7.79 (d, *J* = 7.2 Hz, 1H); chiral HPLC analysis: Chiralcel AD-H column, Hexane/EtOH 96:04, flow rate = 1 mL/min, λ = 254 nm, retention times: **10.4** and 18.4 min.

*Di-tert-butyl 1-(2-acetyl-1-oxo-2,3-dihydro-1H-inden-2-yl)hydrazine-1,2-dicarboxylate* (**13j**) [35]: Slightly yellow oil; [α]_D_^28^ = +41.70, (*c* = 1.0, CHCl_3_, 57% *ee*); ^1^H-NMR (300 MHz, CDCl_3_) δ_H_ *=* 1.38 (br m, 18H), 2.35 (m, *J* = 39.1 Hz, 3H), 3.58 (m, 1H), 4.15 (m, 1H), 6.82 (br s, 1H), 7.34 (d, *J* = 6.7 Hz, 1H), 7.49 (d, *J* = 7.0 Hz, 1H), 7.63 (m, 2H) ppm; chiral HPLC analysis: Chiralcel IA column, Hexane/iPrOH 90:10, flow rate = 1 mL/min, λ = 240 nm, retention times: **19.3** and 24.3 min.

*(S)-Di-tert-butyl 1-[2-(methoxycarbonyl)-1-oxo-2,3-dihydro-1H-tetralone-2-yl]hydrazine-1,2-dicarboxylate* (**13k**) [24]: Slightly yellow oil; [α]_D_^28^ = +6.07 (*c* = 1.2, CHCl_3_, 87% *ee*); ^1^H-NMR (300 MHz, CDCl_3_) δ_H_ = 1.20–1.26 (m, 8H), 1.47 (s, 11H), 2.53–3.09 (m, 4H), 3.82 (s, 3H), 6.22 (br s, 1H), 7.36 (d, *J* = 6.0 Hz, 1H), 7.45 (d, *J* = 6.9 Hz, 1H), 7.69 (d, *J* = 8.3 Hz, 1H), 7.91 (d, *J* = 7.3 Hz, 1H) ppm; chiral HPLC analysis: Chiralcel OD-H column, Hexane/iPrOH 95:05, flow rate = 1 mL/min, λ = 240 nm, retention times: 15.8 and **17.7** min.

*(R)-Di-tert-butyl 1-[2-(ethoxycarbonyl)-1-oxo-2,3-dihydro-1H-tetralone-2-yl]hydrazine-1,2-dicarboxylate* (**13l**) [35]: Slightly yellow sticky oil; [α]_D_^28^ = −11.53 (*c* = 1.2, CHCl_3_, 80% *ee*); ^1^H-NMR (300 MHz, CDCl_3_) δ_H_ = 1.26–1.47 (br m, 21H), 2.67 (dd, *J* = 16.3, 6.5 Hz, 1H), 2.95 (d, *J* = 17.4 Hz, 2H), 3.44 (br s, 1H), 4.23–4.35 (m, 2H), 6.21 (m, 1H), 7.26 (d, *J* = 8.1 Hz, 2H), 7.45 (t, *J* = 7.2 Hz, 1H), 7.89 (d, *J* = 7.6 Hz, 1H) ppm; chiral HPLC analysis: Chiralcel AD-H column, Hexane/iPrOH 85:15, flow rate = 1 mL/min, λ = 240 nm, retention times: 8.6 and **11.4** min.

*(R)-Di-tert-butyl 1-(2-acetyl-1-oxo-1,2,3,4-tetrahydronaphthalen-2-yl)hydrazine-1,2-dicarboxylate* (**13m**) [35]: Dark brown oil; [α]_D_^28^ = −8.23, (*c* = 1.1, CHCl_3_, 27% *ee);* ^1^H-NMR (300 MHz, CDCl_3_) δ_H_ *=* 1.52–1.43 (m, 18H), 2.41 (m, 3H), 2.70 (br s, 2H), 3.13–2.85 (m, 2H), 6.21 (s, 1H), 7.19 (d, *J* = 7.8 Hz, 1H), 7.30 (d, *J* = 3.7 Hz, 1H), 7.46 (t, *J* = 7.4 Hz, 1H), 7.97 (d, *J* = 7.7 Hz, 1H) ppm; chiral HPLC analysis: Chiralcel IA column, Hexane/iPrOH 90:10, flow rate = 1 mL/min, λ = 240 nm, retention times: 19.5 and **22.6** min.

*Di-tert-butyl 1-(1-ethoxy-2-methyl-1,3-dioxo-3-phenylpropan-2-yl)hydrazine-1,2dicarboxylate* (**13n**) [38]: Not isolated; ^1^H-NMR (300 MHz, CDCl_3_) δ_H_ = 1.71–1.19 (m, 24H), 4.33 (dd, *J* = 7.0, 3.1 Hz, 2H), 6.51–5.92 (br s, 1H), 7.56–7.43 (m, 3H), 8.53 (m, 2H) ppm; chiral HPLC analysis: Chiralcel AD-H column, Hexane/iPrOH 90:10, flow rate = 1 mL/min, λ = 210 nm, retention times: = **8.5** and 17.9 min.

## 4. Conclusions

In summary, in this work we have described the synthesis and application of new chiral guanidines derived from benzimidazoles as organocatalysts for the asymmetric electrophilic α-amination of 1,3-dicarbonyl compounds employing di-*t*-butylazodicarboxylate as aminating agent. In general, the new catalysts are synthesized in good yields in a straightforward one-step reaction. In addition, the amination products were obtained in good yields and enantioselectivities varying from moderate to high when 10 mol % of catalysts **1** and **3** were employed. Although a more comprehensive study about the reaction mechanism is necessary, a bifunctional role of the catalyst is postulated.

## Figures and Tables

**Table 1:**
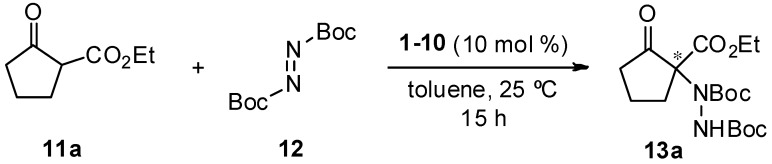
Organocatalyst screening. ^a^

Entry	Catalyst	Conv. (%) ^b^	*ee* (%) ^c^
**1**	**1**	95	91
**2**	**2**	90	66
**3**	**3**	93	90
**4**	**4**	96	78
**5**	**5**	65	40
**6**	**6**	57	33
**7**	**7**	22	Rac
**8**	**8**	85	76
**9**	**9**	40	10
**10**	**10**	~15	27

^a^ Unless otherwise stated, general conditions were **11a** (0.15 mmol), **12** (1.1 equiv.), organocatalyst (10 mol %) in toluene (1 mL) at 25 °C for 15 h. ^b^ Conversions determined by ^1^H-NMR from the crude reaction mixture. ^c^ Determined by chiral HPLC analysis (Daicel Chiralpak IA, see Experimental Section for details).

**Table 2:**
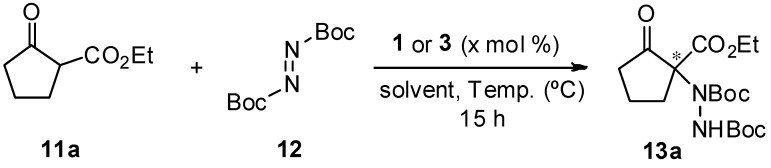
Optimization of the reaction parameters. ^a^

Entry	Catalyst (x mol %)	Solvent	Temp. (°C)	Conv. (%) ^b^	*ee* (%) ^c^
**1**	**1** (10 mol %)	toluene	25	95	91
**2**	**1** (10 mol %)	hexane	25	97	85
**3**	**1** (10 mol %)	Et_2_O	25	99	70
**4**	**1** (10 mol %)	CH_2_Cl_2_	25	99	74
**5**	**1** (10 mol %)	toluene	0	87	92
**6**	**1** (5 mol %)	toluene	25	95	84
**7**	**3** (5 mol %)	toluene	25	90	85

^a^ Unless otherwise stated, general conditions were **11a** (0.15 mmol), **12** (1.1 equiv.), **1** or **3** (5 or 10 mol %) in the corresponding solvent (1 mL) at the corresponding temperature for 15 h. ^b^ Conversions determined by ^1^H-NMR from the crude reaction mixture. ^c^ Determined by chiral HPLC analysis (Daicel Chiralpak IA, see Experimental Section for details).

## Supplementary Materials

General remarks and NMR copies of representative catalysts as well as ^1^H-NMR and HPLC charts of amination compounds are provided.
