# Supplementary material for: Readily Available Chiral Benzimidazoles-Derived Guanidines as Organocatalysts in the Asymmetric α-Amination of 1,3-Dicarbonyl Compounds
Source: Molecules. 2017 Aug 11;22(8):1333. doi: 10.3390/molecules22081333 (PMC6152235; doi:10.3390/molecules22081333)
Supplement: Supplementary file 1 [file molecules-22-01333-s001.pdf]

SUPPORTING INFORMATION

Readily Available Chiral Benzimidazoles-Derived Guanidines as Organocatalysts in the Asymmetric α-Amination of 1,3-Dicarbonyl Compounds

Llorenç Benavent, Francesco Puccetti, Alejandro Baeza * and Melania Gómez-Martínez

Departamento de Química Orgánica and Instituto de Síntesis Orgánica (ISO), Facultad de Ciencias, Universidad de Alicante, Apdo. 99, E-03080 Alicante, Spain; l.benavent@ua.es (L.B.); francesco.puccetti@stud.unifi.it (F.P.); melania.gomez@ua.es (M.G.-M.)

* Correspondence: alex.baeza@ua.es; Tel.: +34-965-902-888

Azodicarboxylate tests and NLE experiments 2

^{1}H and ^{13}CNMR Spectra of Catalysts 3

^{1}H NMR Spectra of Chiral Amination Products 13

HPLC Chromatograms of Chiral Amination Products 19

Azodicarboxylate tests

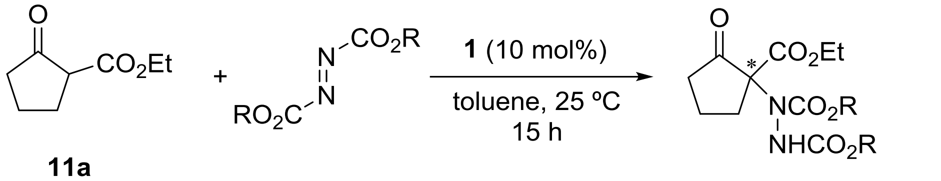

11a

R = Et, >95% conv. 55% *ee*
R = Bn, ≈70% conv. 64% *ee*

Non Linear Effects Experiments

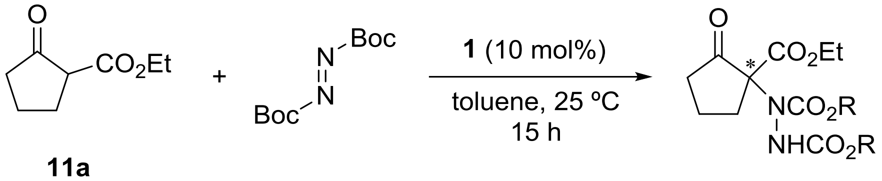

11a

%ee **1**	%*ee* theor.	%*ee* observ.
0	0	0
30	28	33
50	46.5	49
80	74	73
99	91	91

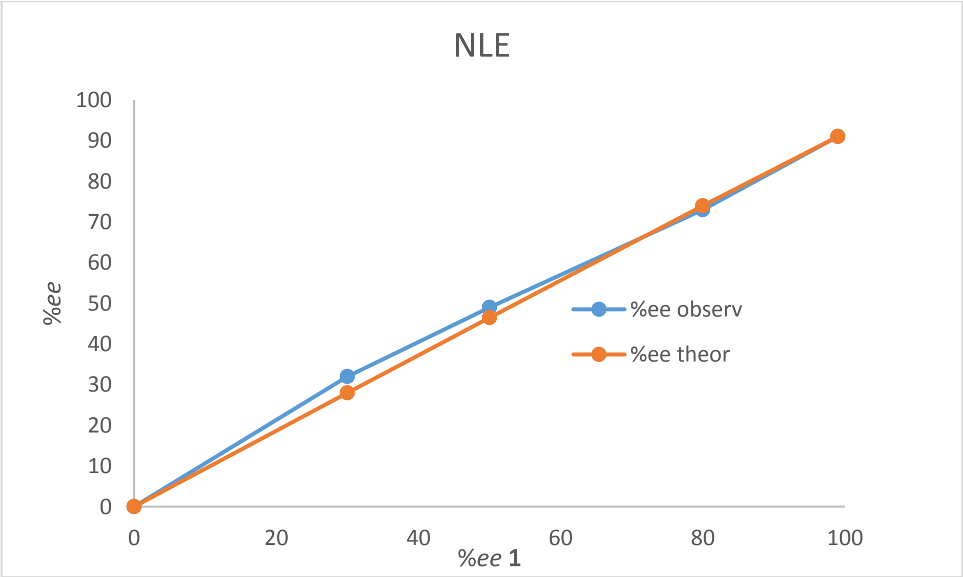

^{1}H and ^{13}C NMR Spectra of Catalysts

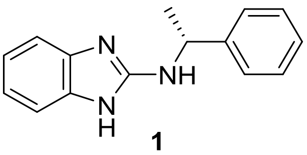

1

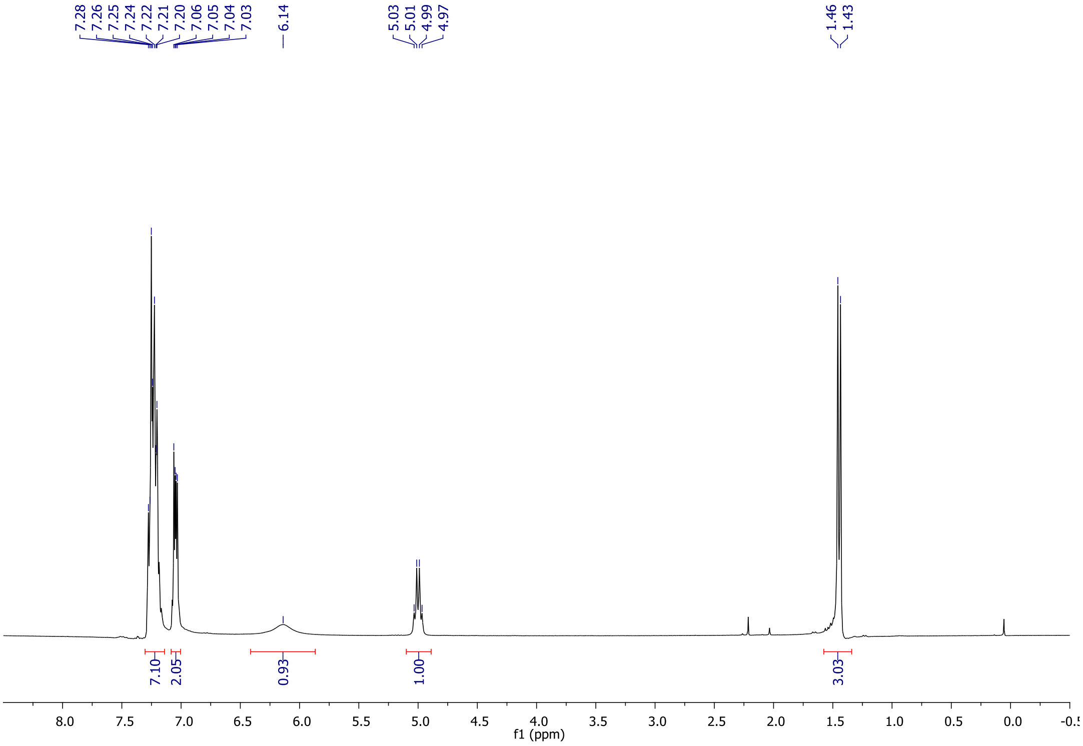

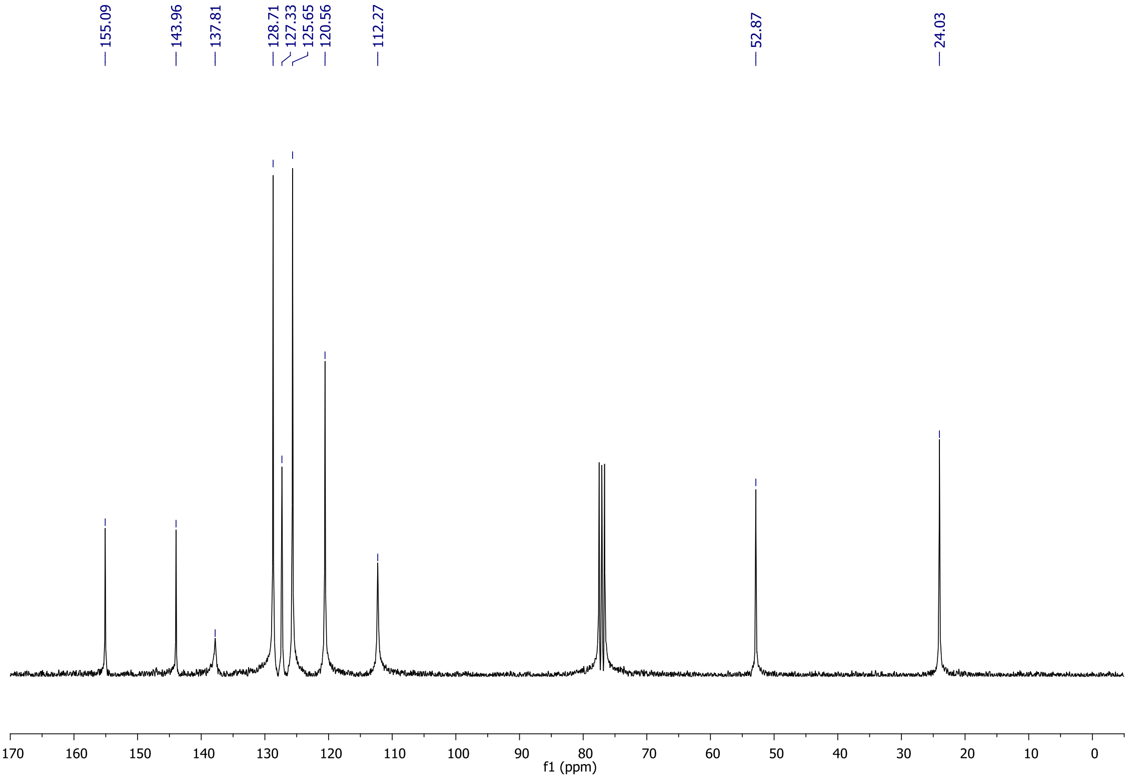

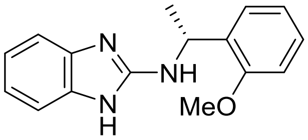
N
NH
N
H
MeO

2

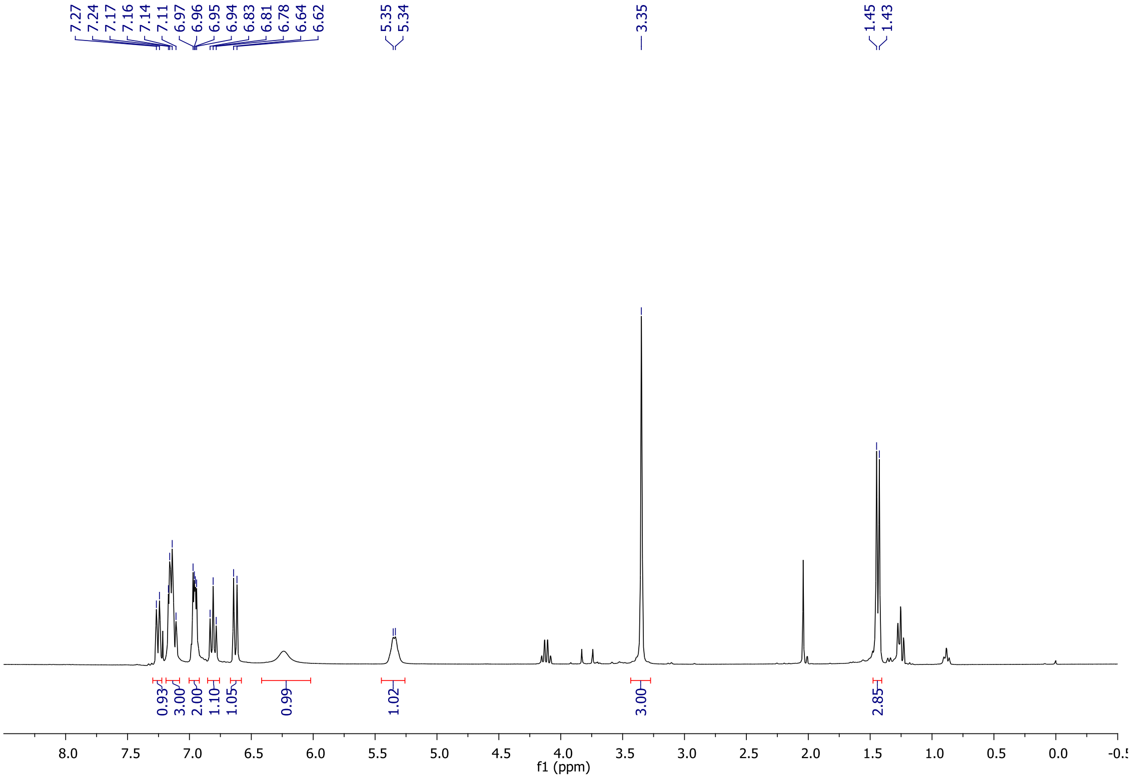
7.27
7.24
7.17
7.16
7.14
7.11
6.97
6.96
6.95
6.94
6.83
6.81
6.78
6.64
6.62
5.35
5.34
3.35
1.45
1.43
0.93
3.00
2.00
1.10
1.05
0.99
1.02
3.00
2.85
8.0 7.5 7.0 6.5 6.0 5.5 5.0 4.5 4.0 3.5 3.0 2.5 2.0 1.5 1.0 0.5 0.0
f1 (ppm)

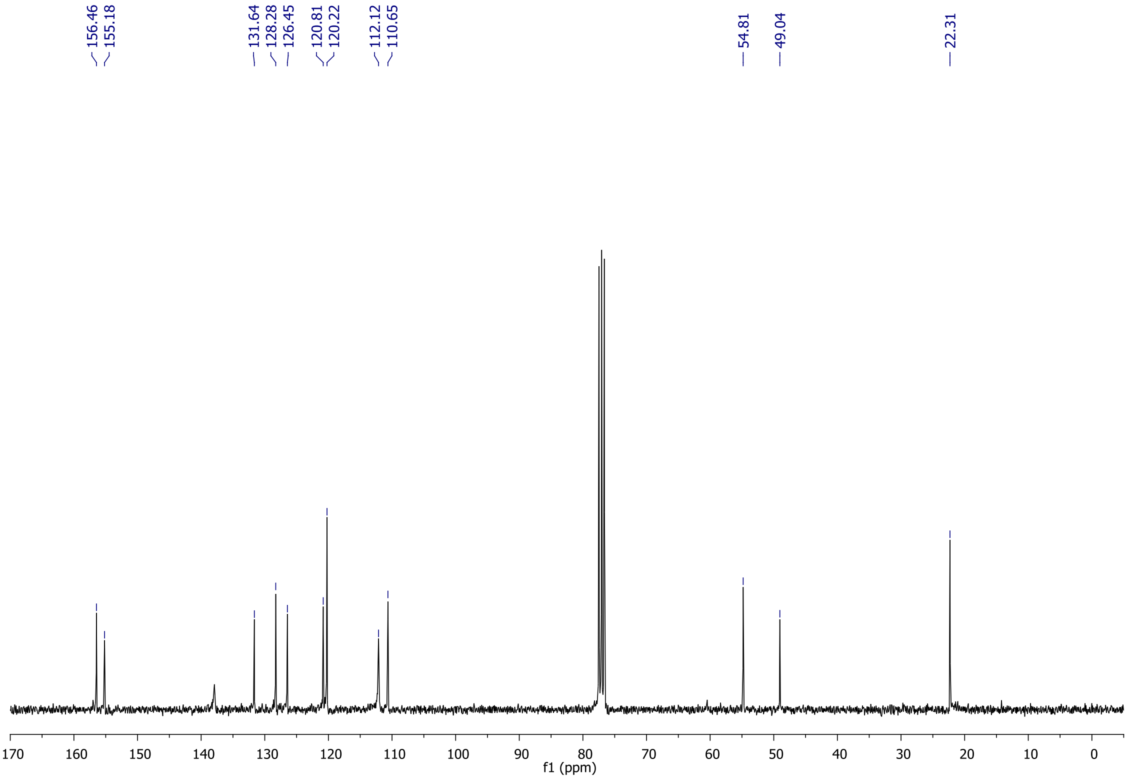
156.46
155.18
131.64
128.28
126.45
120.81
120.22
112.12
110.65
54.81
49.04
22.31
170 160 150 140 130 120 110 100 90 80 70 60 50 40 30 20 10 0
f1 (ppm)

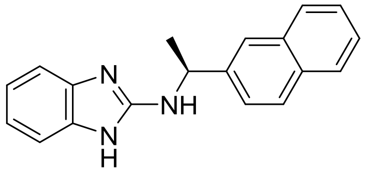
N
NH
N
H

3

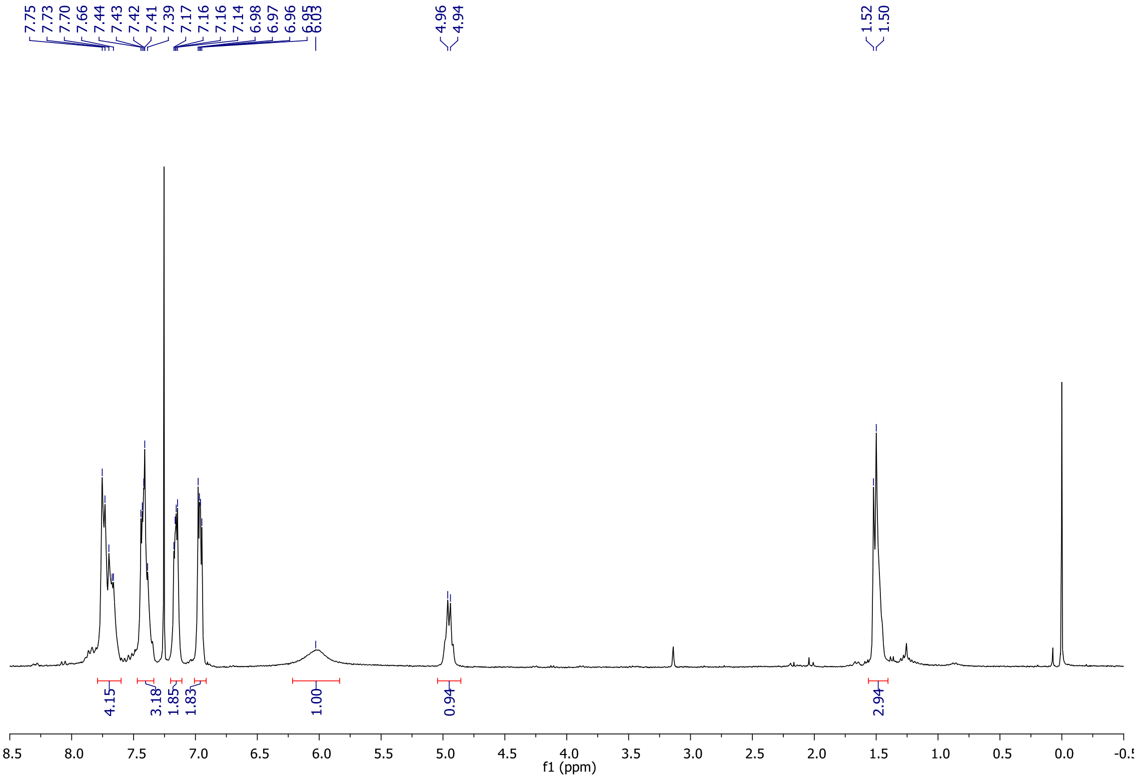
7.75 7.73 7.70 7.66 7.44 7.43 7.42 7.41 7.39 7.17 7.16 7.16 7.14 6.98 6.97 6.96 6.95 6.03
4.96 4.94
1.52 1.50
4.15 3.18 1.85 1.83 1.00 0.94 2.94
8.5 8.0 7.5 7.0 6.5 6.0 5.5 5.0 4.5 4.0 3.5 3.0 2.5 2.0 1.5 1.0 0.5 0.0
f1 (ppm)

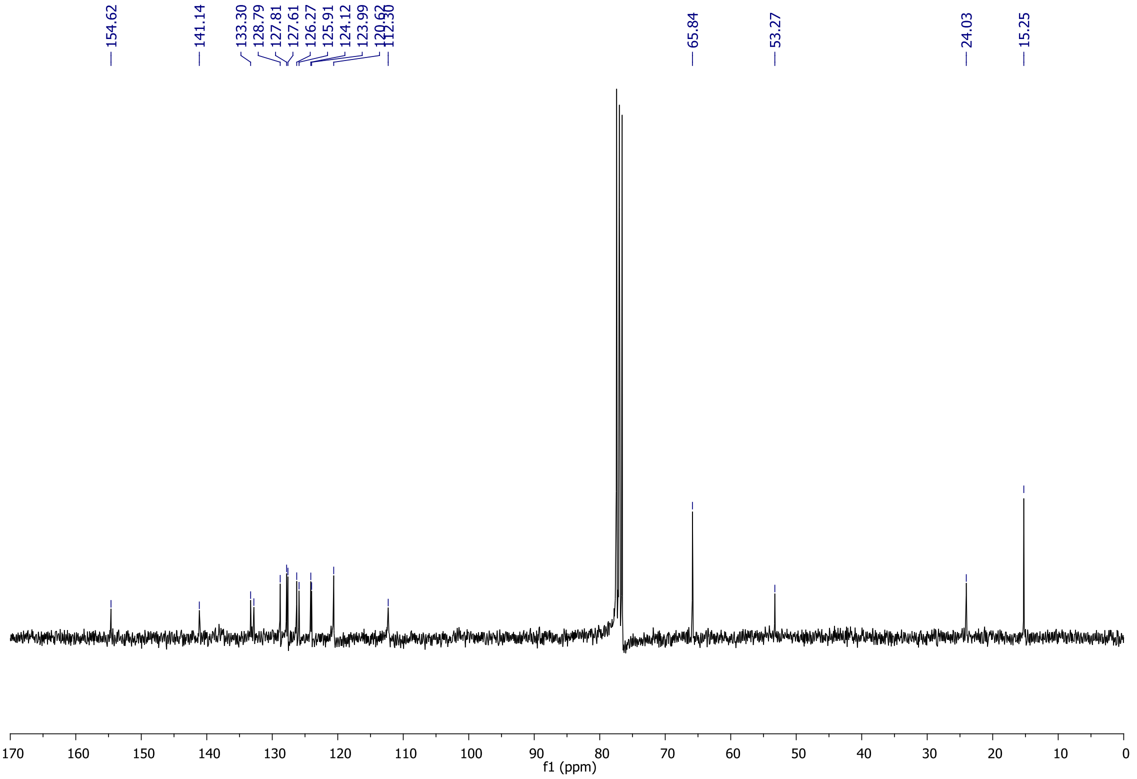
154.62
141.14
133.30 128.79 127.81 127.61 126.27 125.91 124.12 123.99 120.62 112.30
65.84
53.27
24.03
15.25
170 160 150 140 130 120 110 100 90 80 70 60 50 40 30 20 10 0
f1 (ppm)

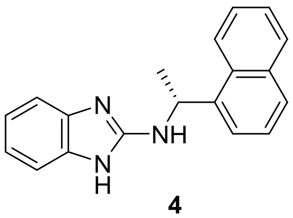

4

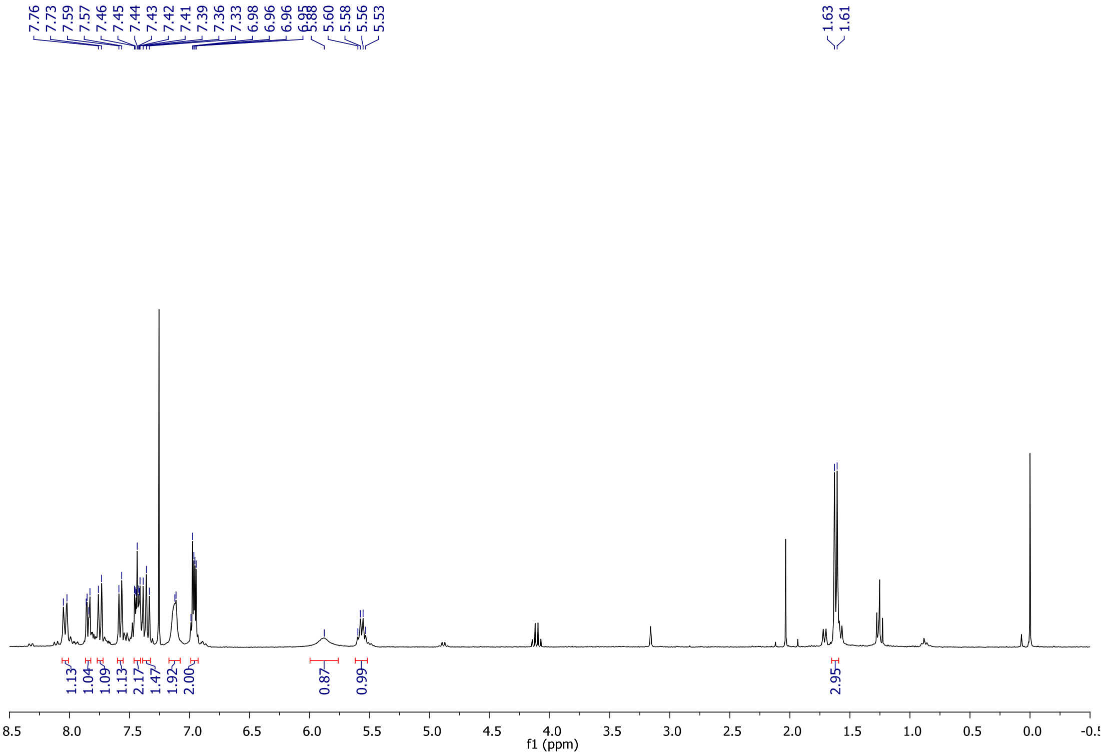
7.76
7.73
7.59
7.57
7.46
7.45
7.44
7.43
7.42
7.41
7.39
7.36
7.33
6.98
6.96
6.96
6.95
5.88
5.60
5.58
5.56
5.53
1.63
1.61
1.13
1.04
1.09
1.13
2.17
1.47
1.92
2.00
0.87
0.99
2.95
f1 (ppm)

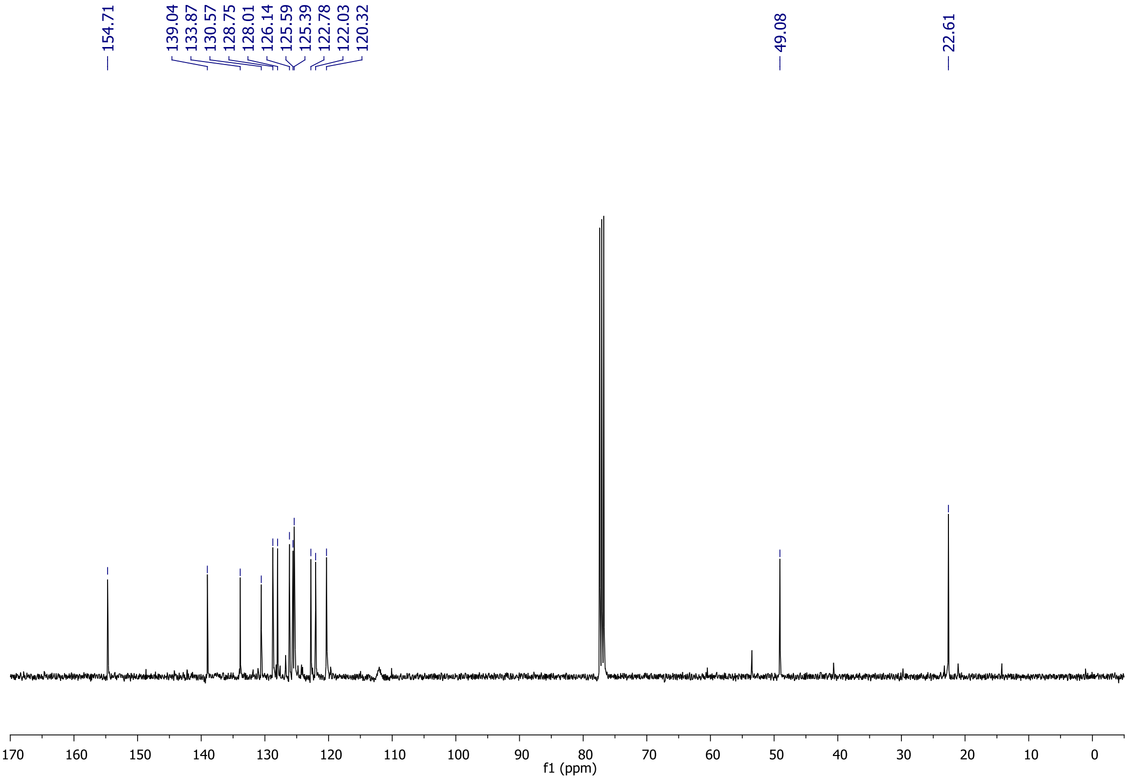
154.71
139.04
133.87
130.57
128.75
128.01
126.14
125.59
125.39
122.78
122.03
120.32
49.08
22.61
f1 (ppm)

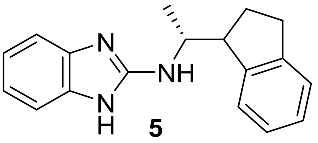

5

^{1}H NMR (300 MHz, $CDCl_3$) δ 7.37 – 7.26 (m, 9H), 7.26 – 7.22 (m, 6H), 7.17 (ddd, J = 12.9, 8.1, 2.2 Hz, 5H), 7.09 – 7.02 (m, 6H), 5.39 (t, J = 7.1 Hz, 3H), 5.27 (d, J = 20.1 Hz, 3H), 2.98 (ddd, J = 12.3, 8.8, 3.9 Hz, 3H), 2.85 (dt, J = 15.8, 8.0 Hz, 4H), 2.72 – 2.59 (m, 4H), 1.91 (ddd, J = 16.0, 12.8, 8.4 Hz, 4H).

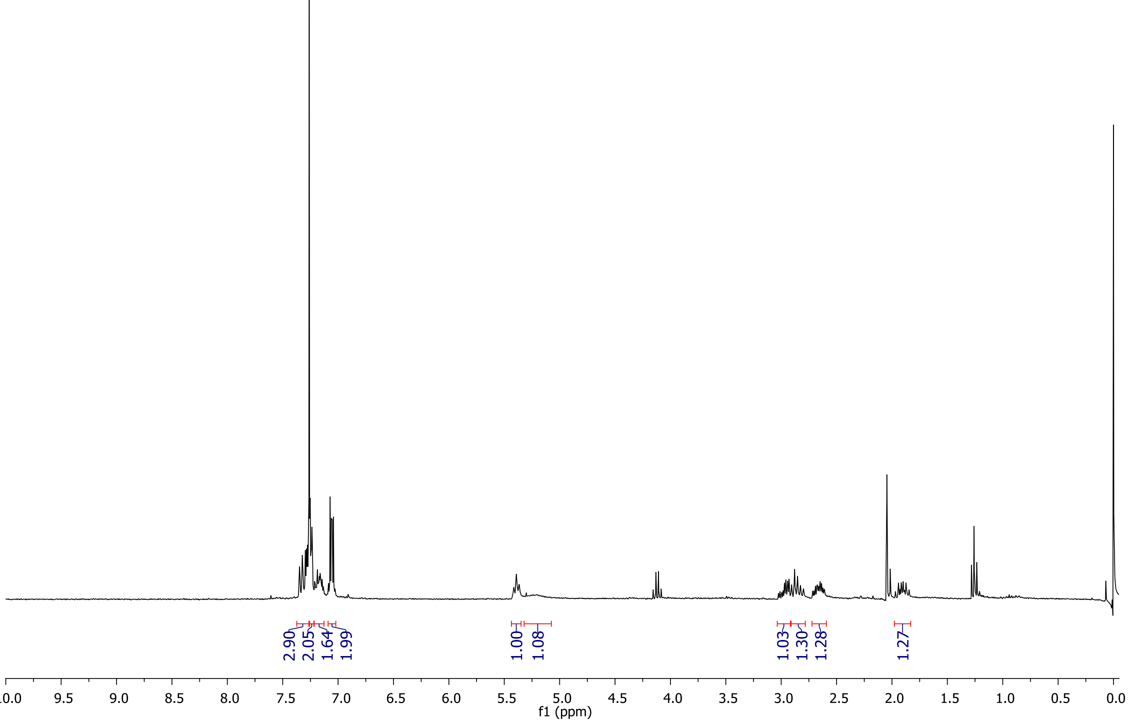

^{13}C NMR (101 MHz, $CDCl_3$) δ 154.35, 143.12, 142.76, 128.11, 126.73, 124.89, 123.88, 120.98, 112.10, 58.52, 34.27, 30.01.

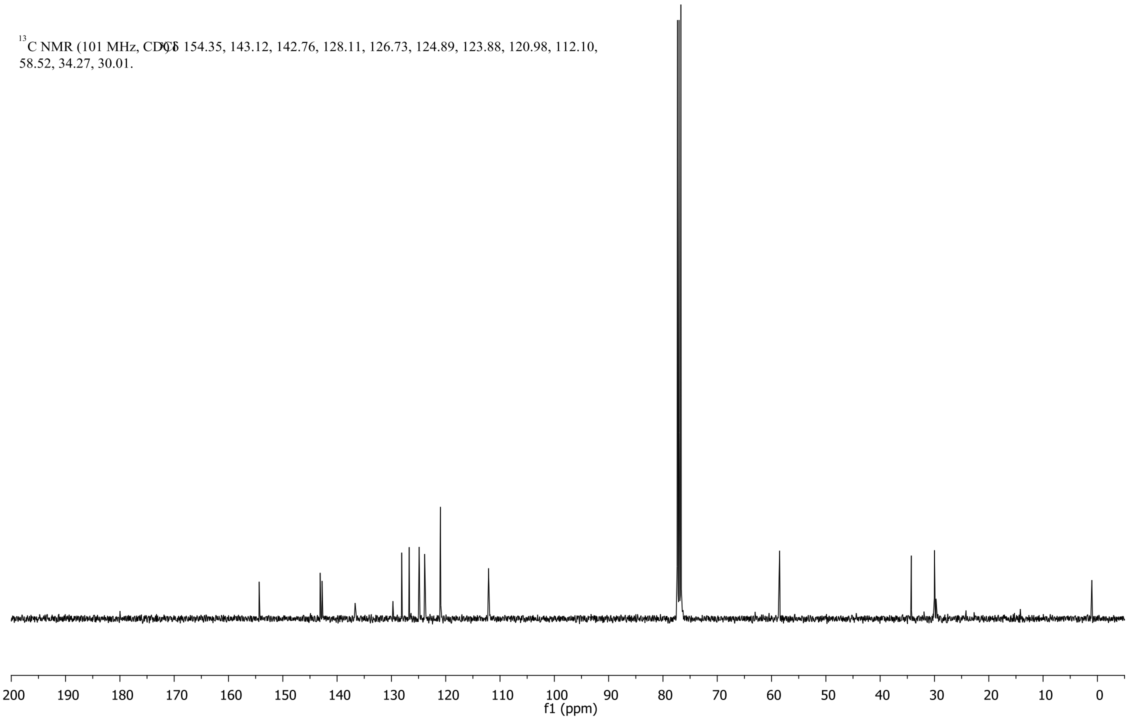

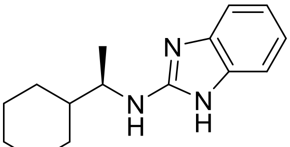

6

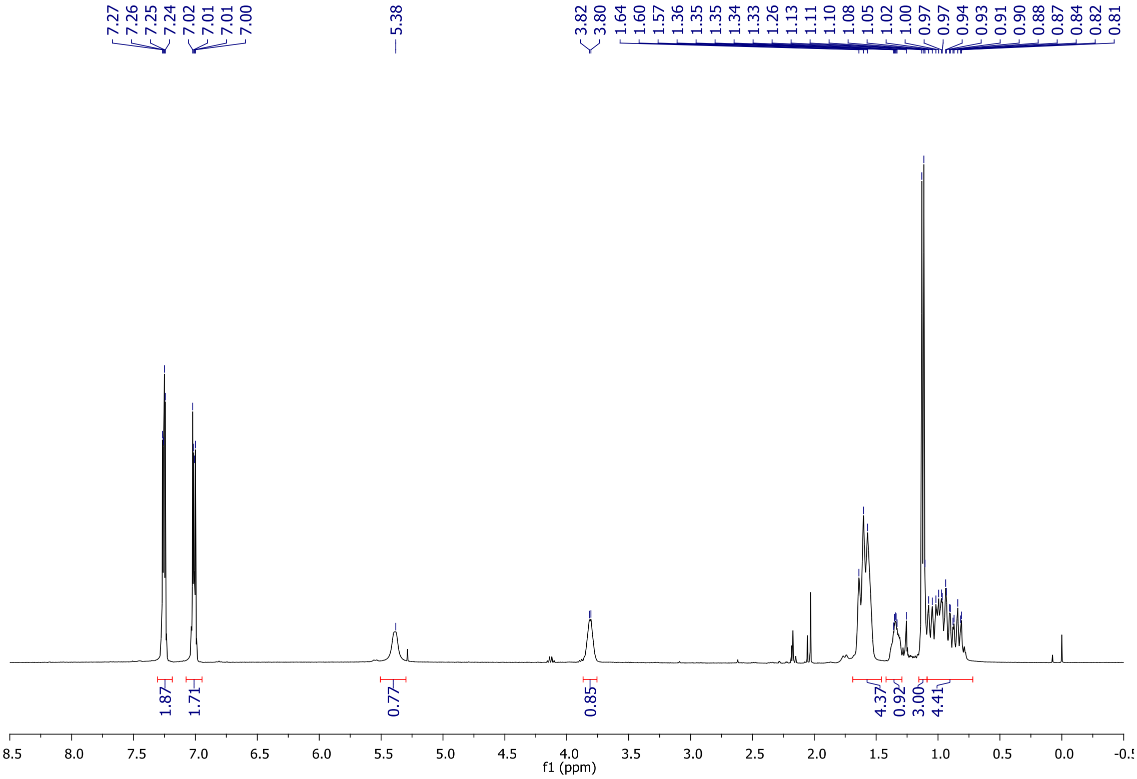
7.27
7.26
7.25
7.24
7.02
7.01
7.01
7.00
5.38
3.82
3.80
1.64
1.60
1.57
1.36
1.35
1.35
1.34
1.33
1.26
1.13
1.11
1.10
1.08
1.05
1.02
1.00
0.97
0.97
0.94
0.93
0.91
0.90
0.88
0.87
0.84
0.82
0.81
1.87
1.71
0.77
0.85
4.37
0.92
3.00
4.41
f1 (ppm)

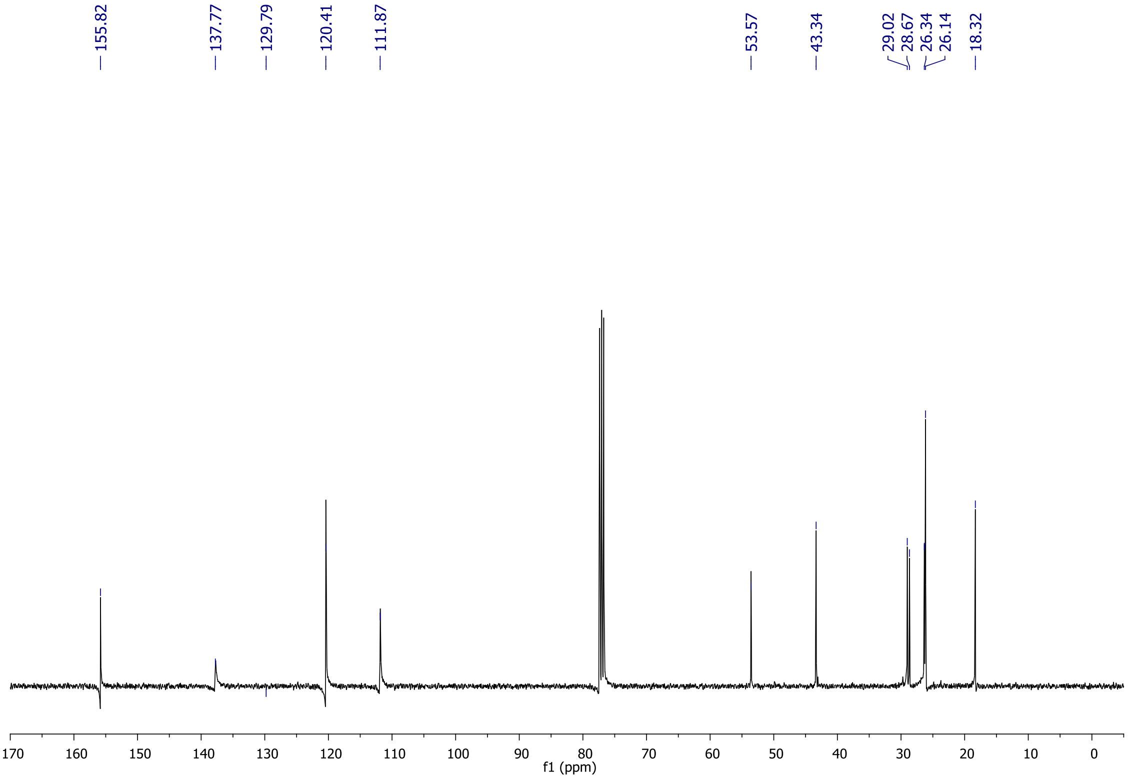
155.82
137.77
129.79
120.41
111.87
53.57
43.34
29.02
28.67
26.34
26.14
18.32
f1 (ppm)

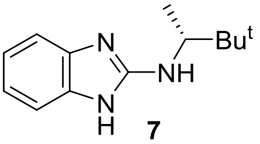

7

^{1}H NMR (300 MHz, CDCl$_3$) δ 7.25 (td, J = 6.1, 2.6 Hz, 2H), 7.01 (dt, J = 10.2, 5.1 Hz, 2H), 5.31 (s, 1H), 3.80 (s, 1H), 1.08 (d, J = 11.8 Hz, 3H), 0.79 (s, 9H).

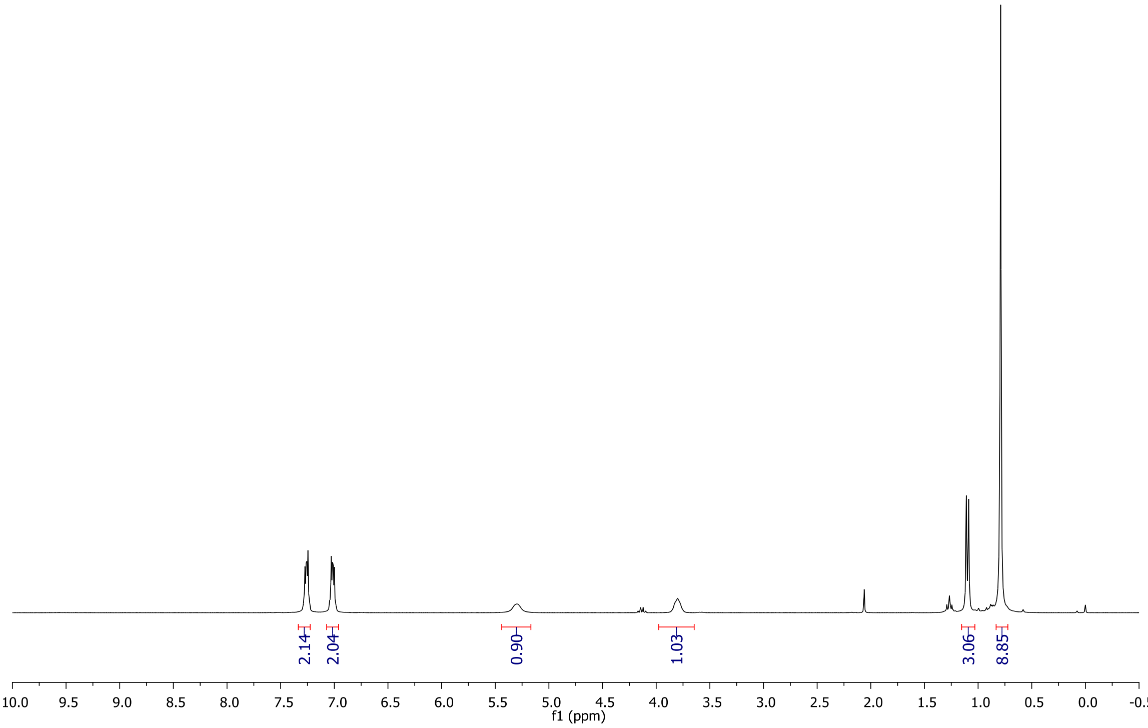

^{13}C NMR (75 MHz, CDCl$_3$) δ 156.39, 120.39, 111.88, 57.44, 34.40, 26.11, 16.74.

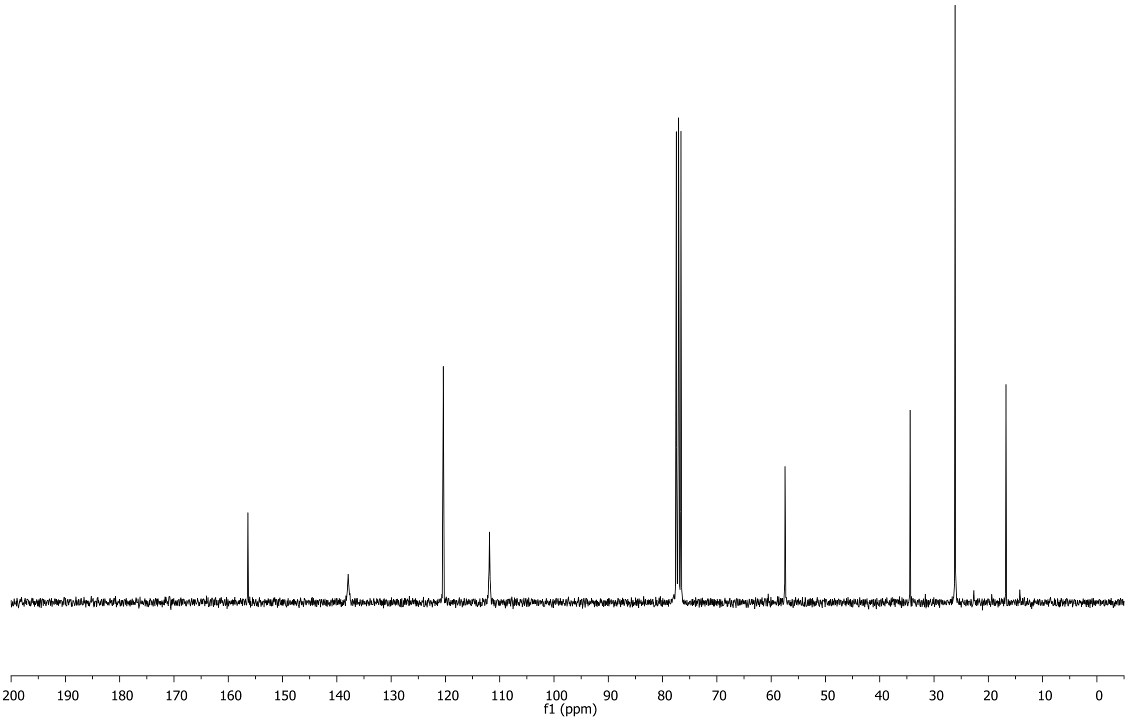

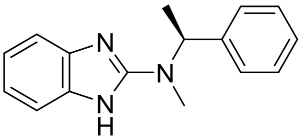

8

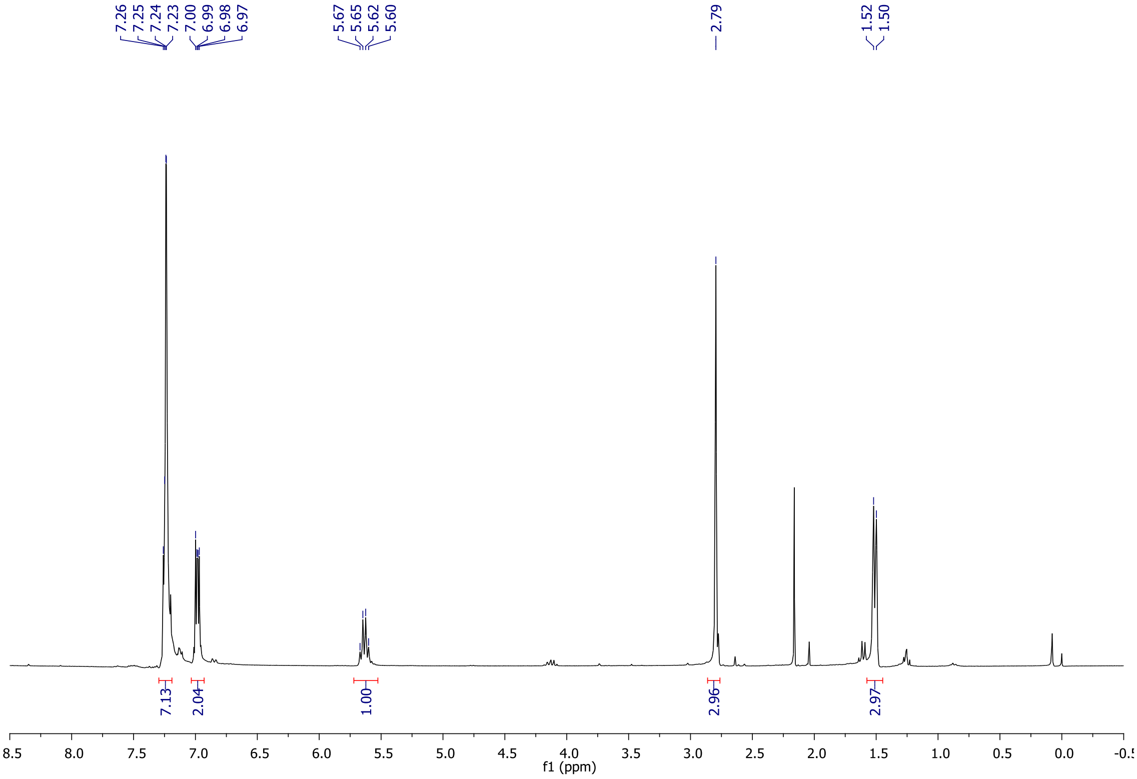
7.26
7.25
7.24
7.23
7.00
6.99
6.98
6.97
5.67
5.65
5.62
5.60
2.79
1.52
1.50
7.13
2.04
1.00
2.96
2.97
8.5 8.0 7.5 7.0 6.5 6.0 5.5 5.0 4.5 4.0 3.5 3.0 2.5 2.0 1.5 1.0 0.5 0.0
f1 (ppm)

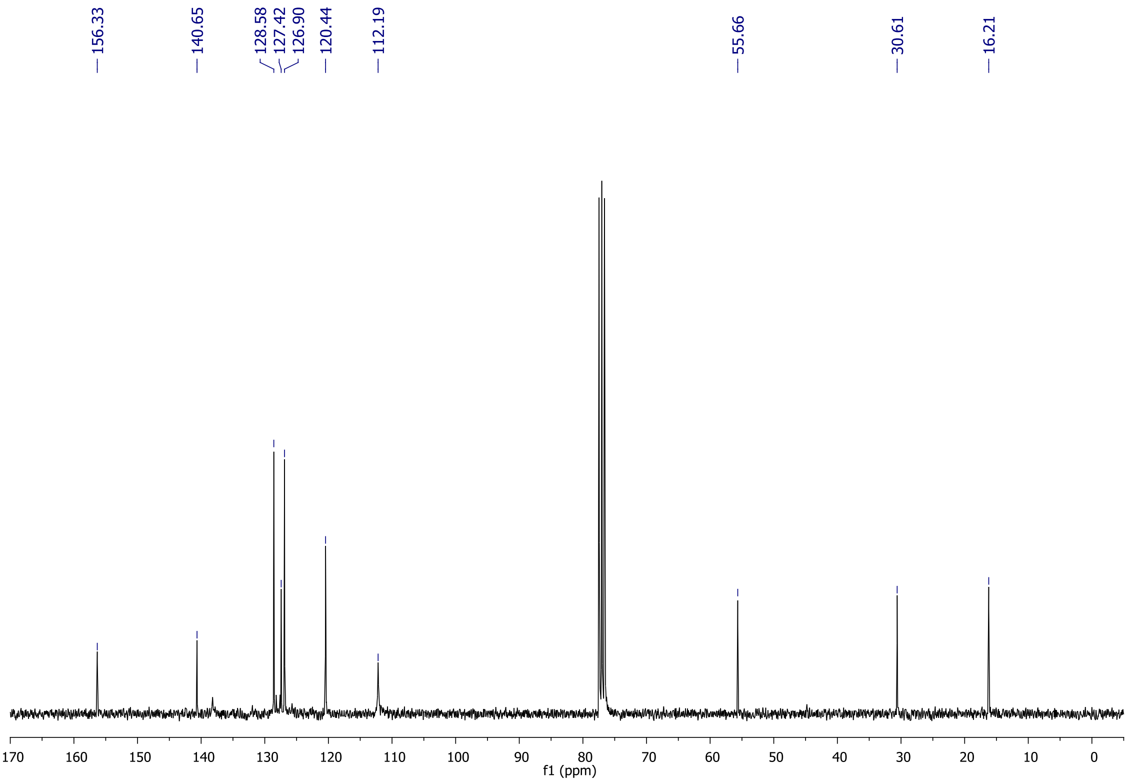
156.33
140.65
128.58
127.42
126.90
120.44
112.19
55.66
30.61
16.21
170 160 150 140 130 120 110 100 90 80 70 60 50 40 30 20 10 0
f1 (ppm)

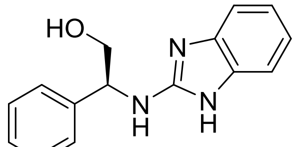
HO
N
N
H
N
H

9

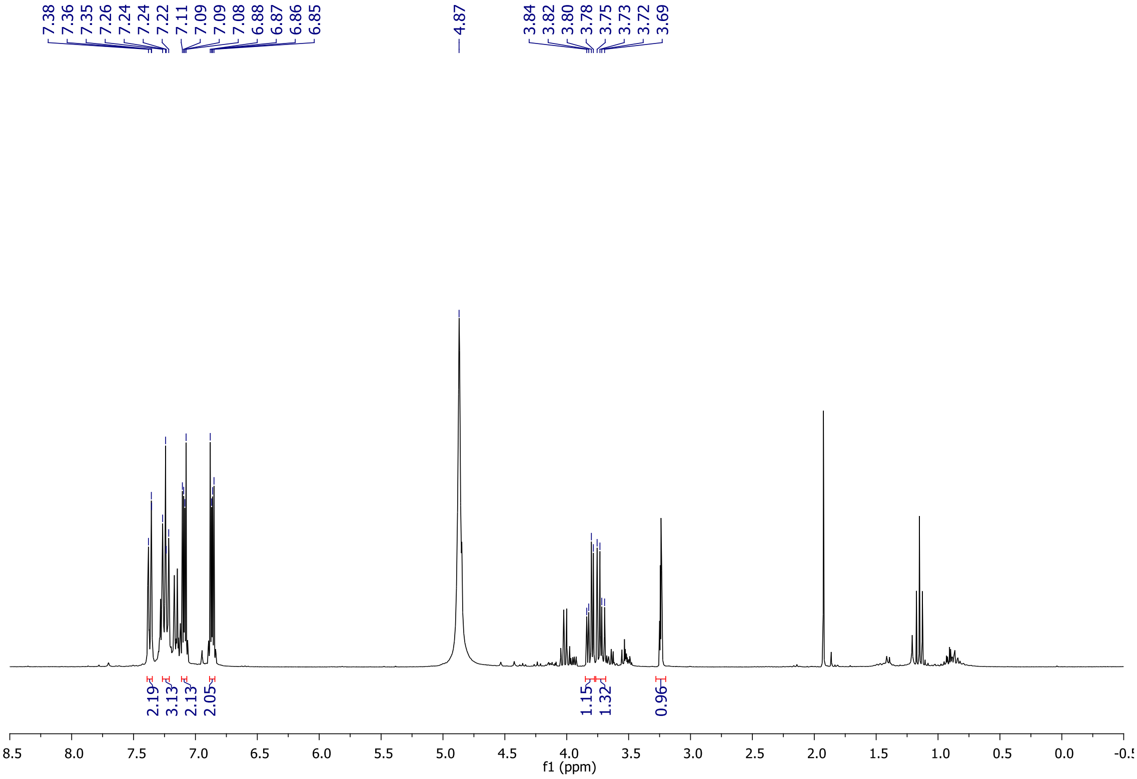
7.38 7.36 7.35 7.26 7.24 7.24 7.22 7.11 7.09 7.09 7.08 6.88 6.87 6.86 6.85
4.87
3.84 3.82 3.80 3.78 3.75 3.73 3.72 3.69
2.19 3.13 2.13 2.05
1.15 1.32
0.96
f1 (ppm)

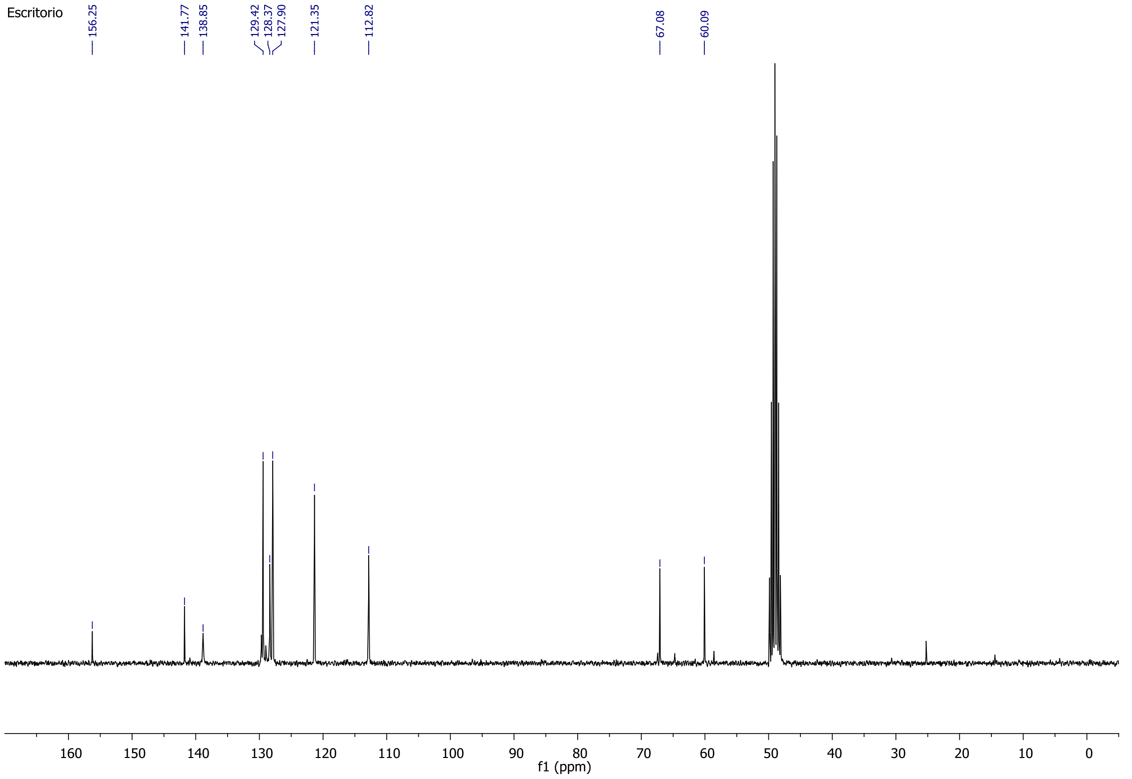
Escritorio
156.25
141.77 138.85
129.42 128.37 127.90
121.35
112.82
67.08
60.09
f1 (ppm)

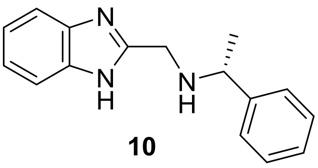

10

^{1}H NMR (300 MHz, $CDCl_3$) δ 7.59 – 7.49 (m, 5H), 7.39 – 7.14 (m, 19H), 3.90 (d, J = 2.1 Hz, 4H), 3.76 (d, J = 3.3 Hz, 3H), 1.37 (d, J = 6.6 Hz, 7H).

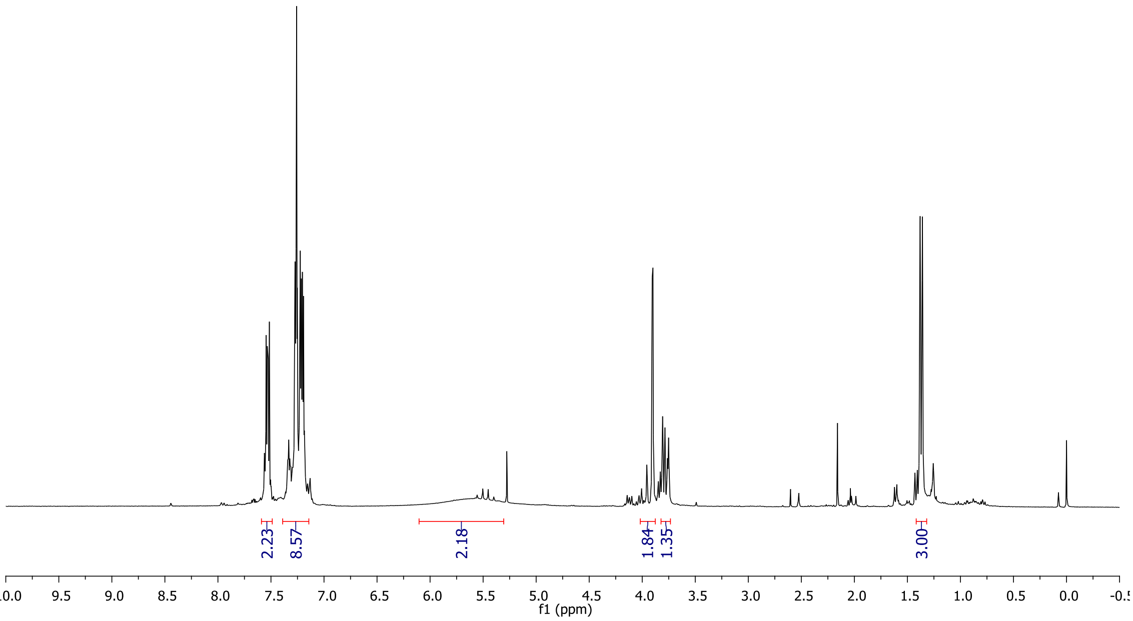

^{13}C NMR (75 MHz, $CDCl_3$) δ 153.93, 144.21, 138.34, 128.62, 127.33, 126.55, 122.36, 114.90, 77.48, 77.06, 76.64, 58.14, 45.34, 23.86.

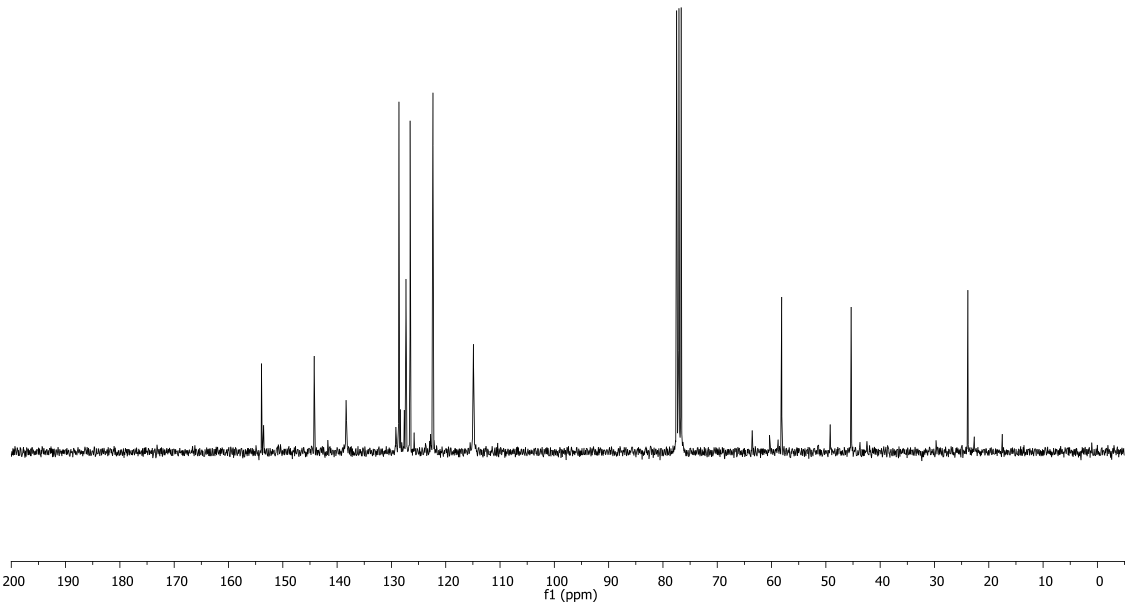

^{1}H NMR Spectra of Chiral Amination Products

Only copy of ^{1}H NMR of those enantioenriched products (≥ 20% *ee*) are provided.

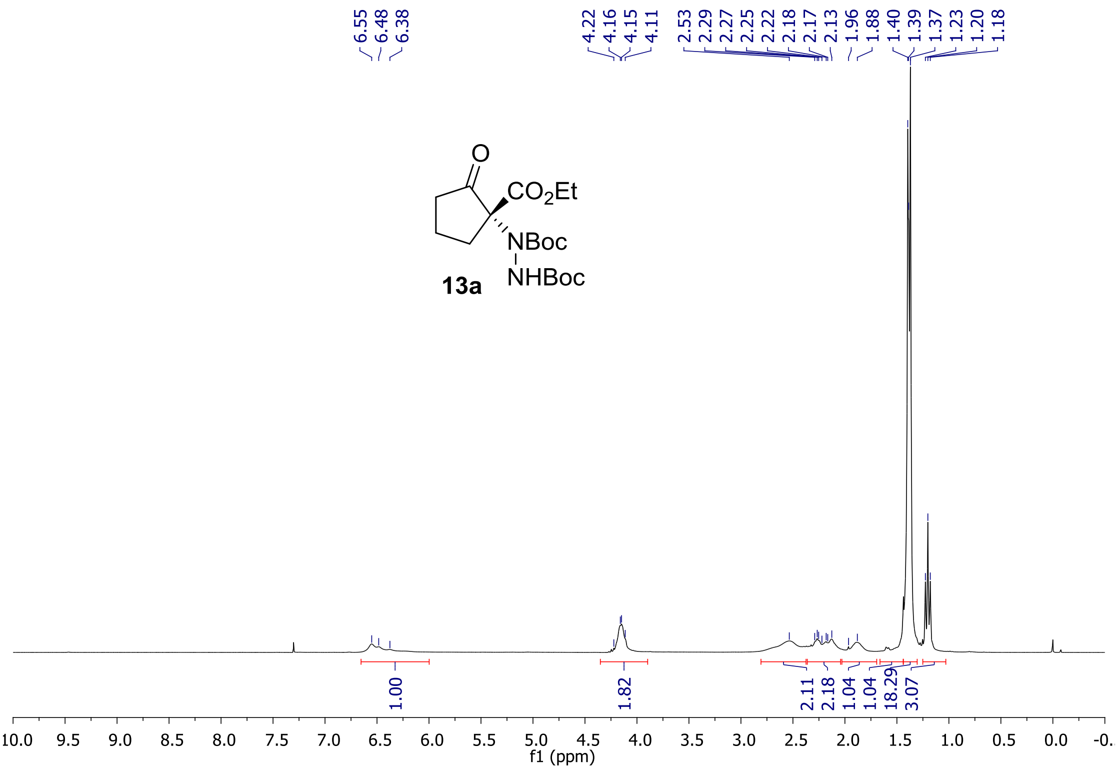

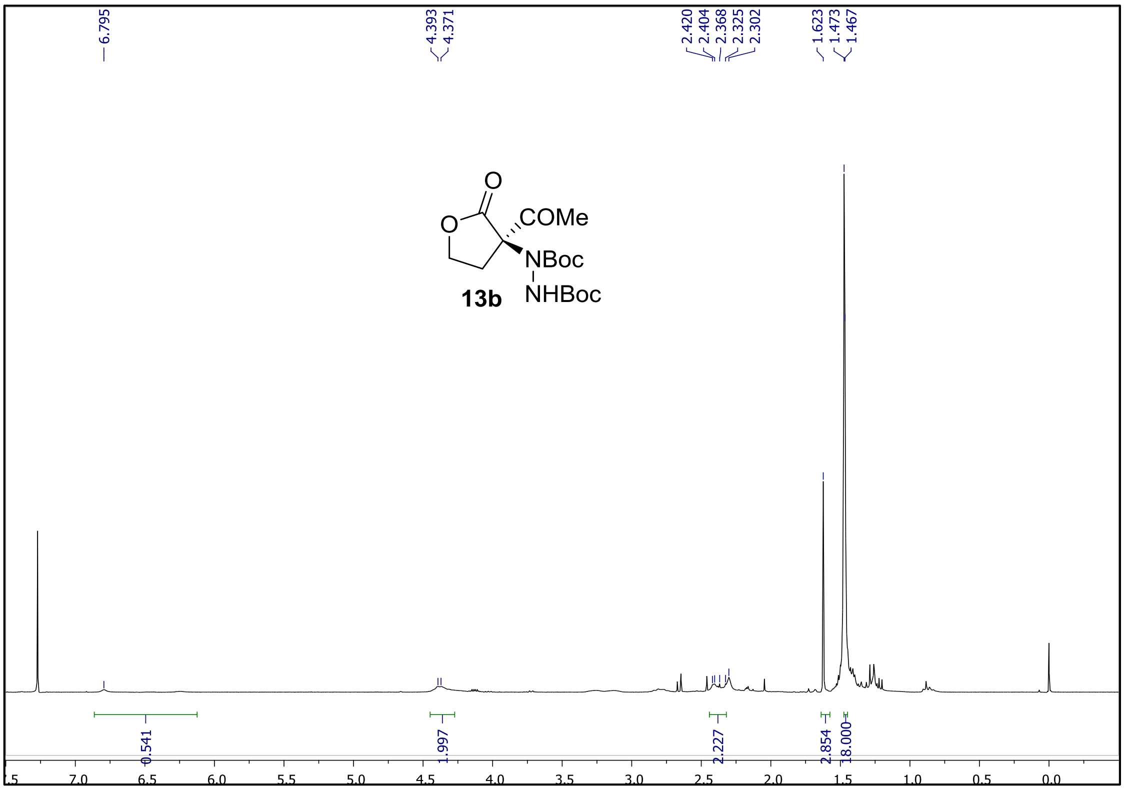

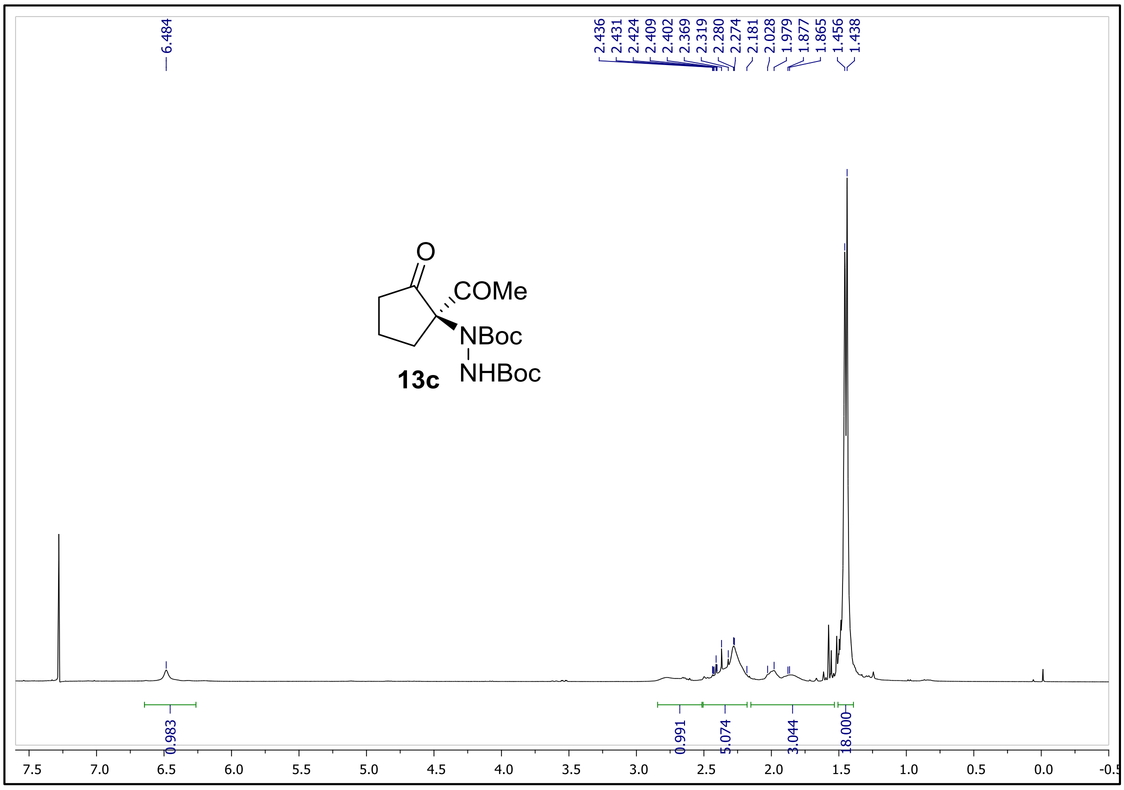
6.484
2.436 2.431 2.424 2.409 2.402 2.369 2.319 2.280 2.274 2.181 2.028 1.979 1.877 1.865 1.456 1.438
O
COMe
NBoc
NHBoc
13c
0.983
0.991
5.074
3.044
18.000
7.5 7.0 6.5 6.0 5.5 5.0 4.5 4.0 3.5 3.0 2.5 2.0 1.5 1.0 0.5 0.0

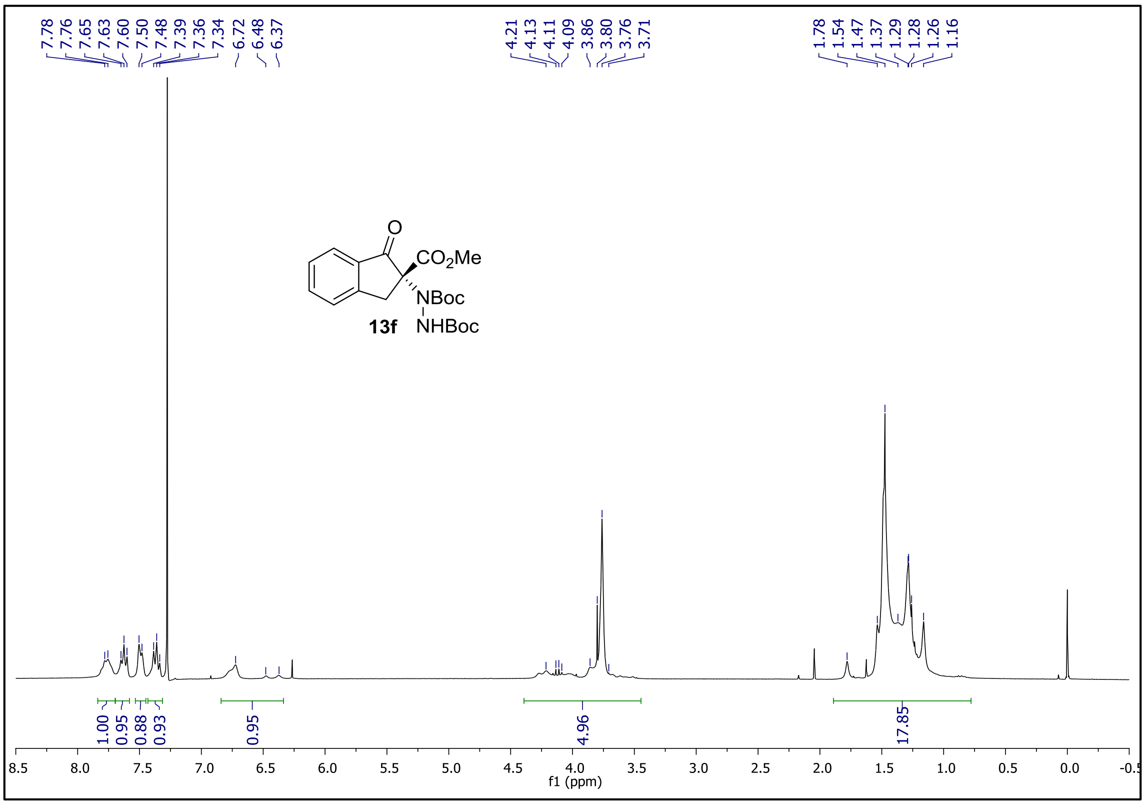
7.78 7.76 7.65 7.63 7.60 7.50 7.48 7.39 7.36 7.34 6.72 6.48 6.37
4.21 4.13 4.11 4.09 3.86 3.80 3.76 3.71
1.78 1.54 1.47 1.37 1.29 1.28 1.26 1.16
O
CO2Me
NBoc
NHBoc
13f
1.00
0.95
0.88
0.93
0.95
4.96
17.85
8.5 8.0 7.5 7.0 6.5 6.0 5.5 5.0 4.5 4.0 3.5 3.0 2.5 2.0 1.5 1.0 0.5 0.0
f1 (ppm)

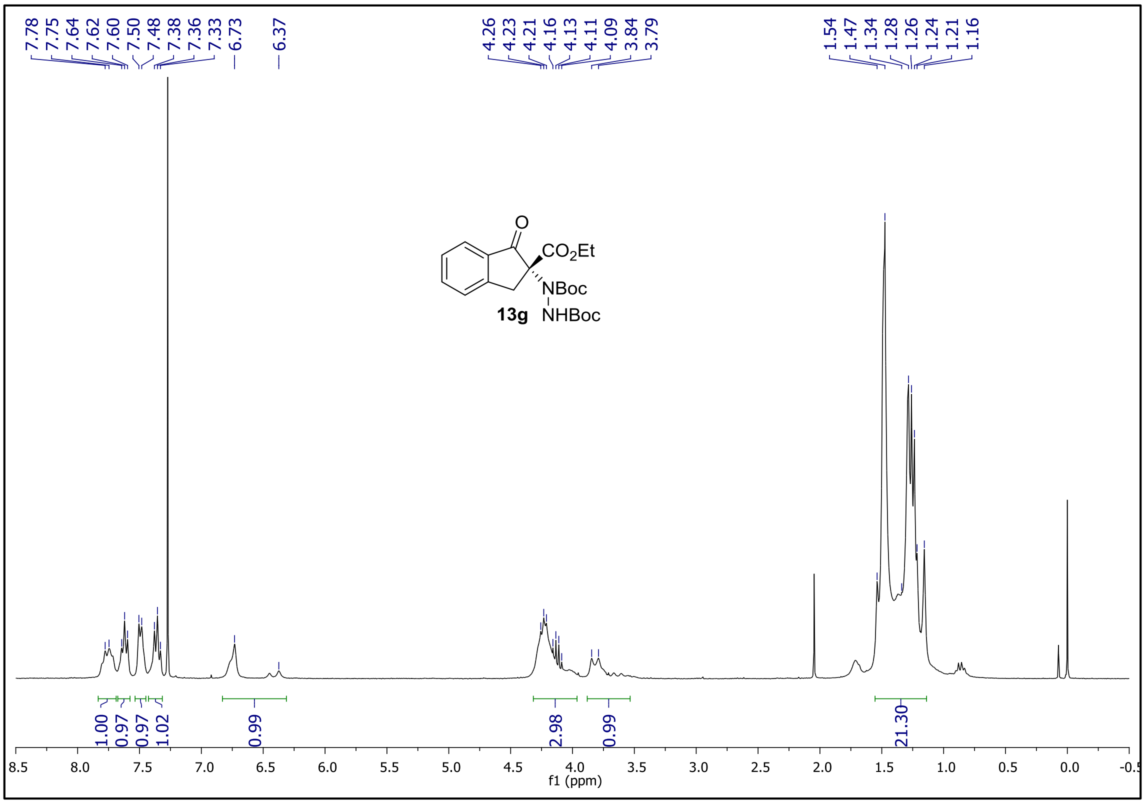
7.78 7.75 7.64 7.62 7.60 7.50 7.48 7.38 7.36 7.33 6.73 6.37
4.26 4.23 4.21 4.16 4.13 4.11 4.09 3.84 3.79
1.54 1.47 1.34 1.28 1.26 1.24 1.21 1.16
O
CO2Et
NBoc
13g
NHBoc
1.00 0.97 0.97 1.02 0.99 2.98 0.99 21.30
8.5 8.0 7.5 7.0 6.5 6.0 5.5 5.0 4.5 4.0 3.5 3.0 2.5 2.0 1.5 1.0 0.5 0.0
f1 (ppm)

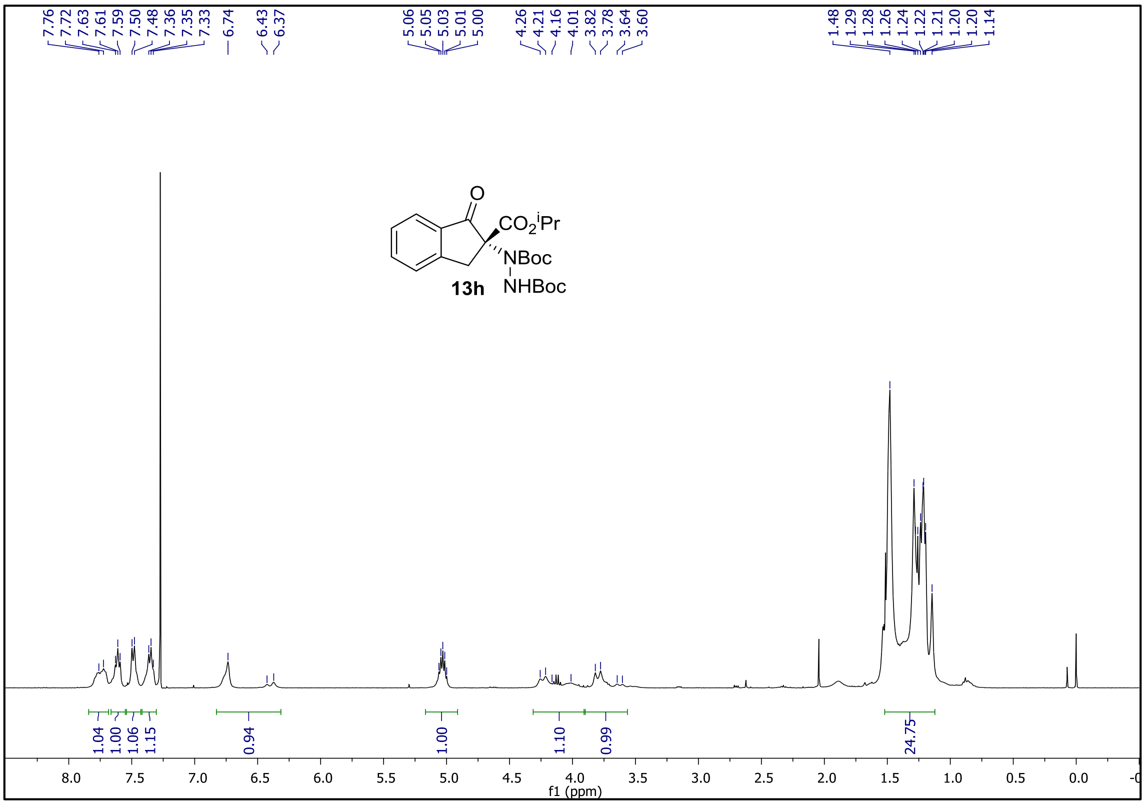
7.76 7.72 7.63 7.61 7.59 7.50 7.48 7.36 7.35 7.33 6.74 6.43 6.37
5.06 5.05 5.03 5.01 5.00 4.26 4.21 4.16 4.01 3.82 3.78 3.64 3.60
1.48 1.29 1.28 1.26 1.24 1.22 1.21 1.20 1.20 1.14
O
CO2iPr
NBoc
13h
NHBoc
1.04 1.00 1.06 1.15 0.94 1.00 1.10 0.99 24.75
8.0 7.5 7.0 6.5 6.0 5.5 5.0 4.5 4.0 3.5 3.0 2.5 2.0 1.5 1.0 0.5 0.0
f1 (ppm)

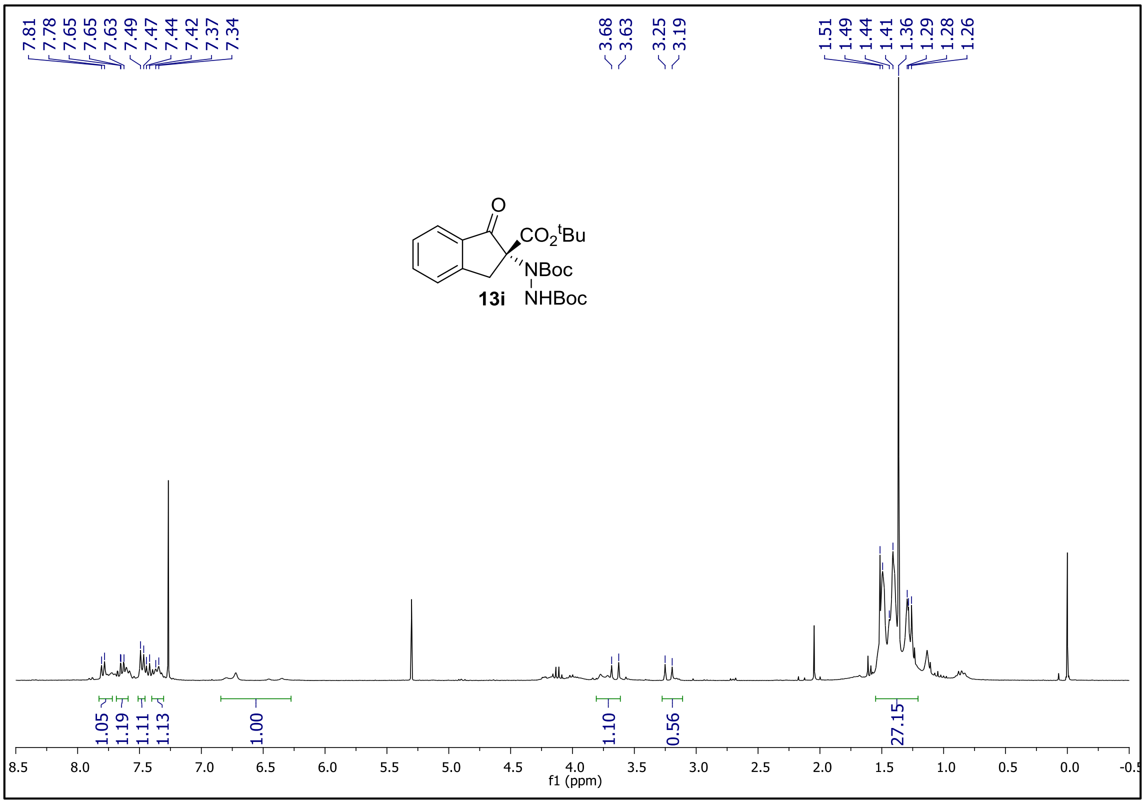
7.81 7.78 7.65 7.63 7.49 7.47 7.44 7.42 7.37 7.34
3.68 3.63 3.25 3.19
1.51 1.49 1.44 1.41 1.36 1.29 1.28 1.26
O
CO2tBu
NBoc
NHBoc
13i
1.05 1.19 1.11 1.13 1.00 1.10 0.56 27.15
f1 (ppm)

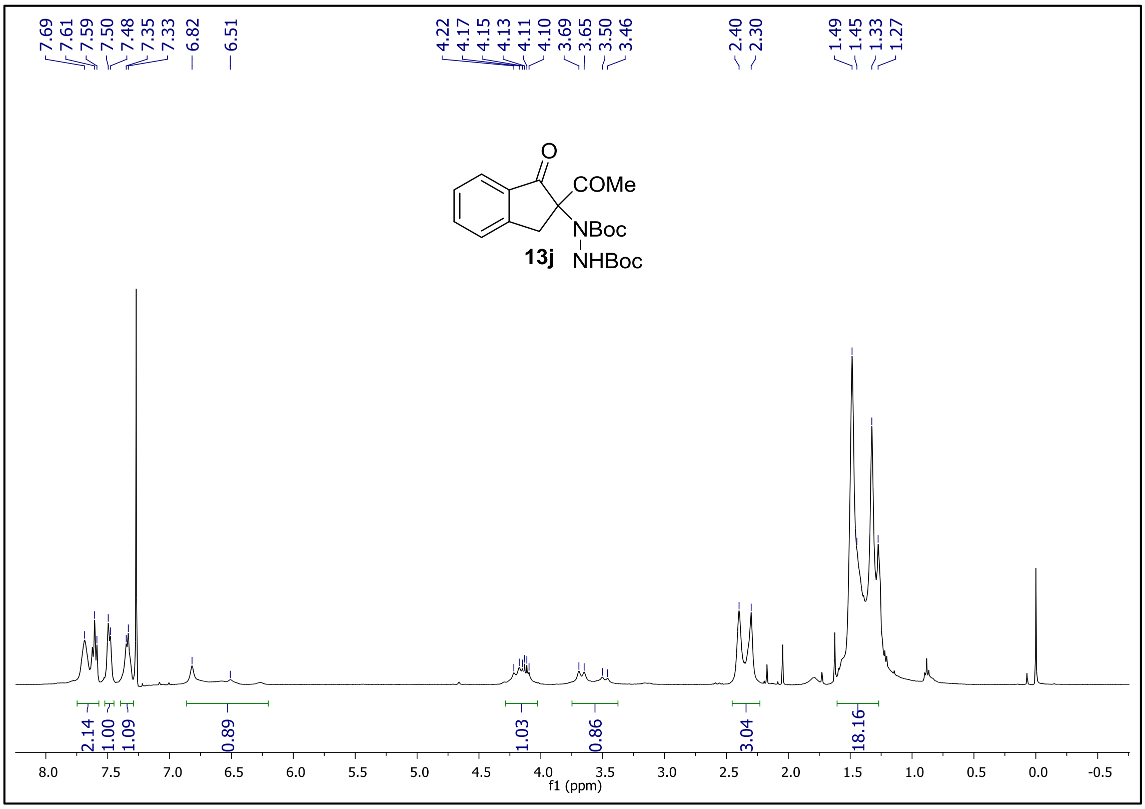
7.69 7.61 7.59 7.50 7.48 7.35 7.33 6.82 6.51
4.22 4.17 4.15 4.13 4.11 4.10 3.69 3.65 3.50 3.46
2.40 2.30
1.49 1.45 1.33 1.27
O
COMe
NBoc
NHBoc
13j
2.14 1.00 1.09 0.89 1.03 0.86 3.04 18.16
f1 (ppm)

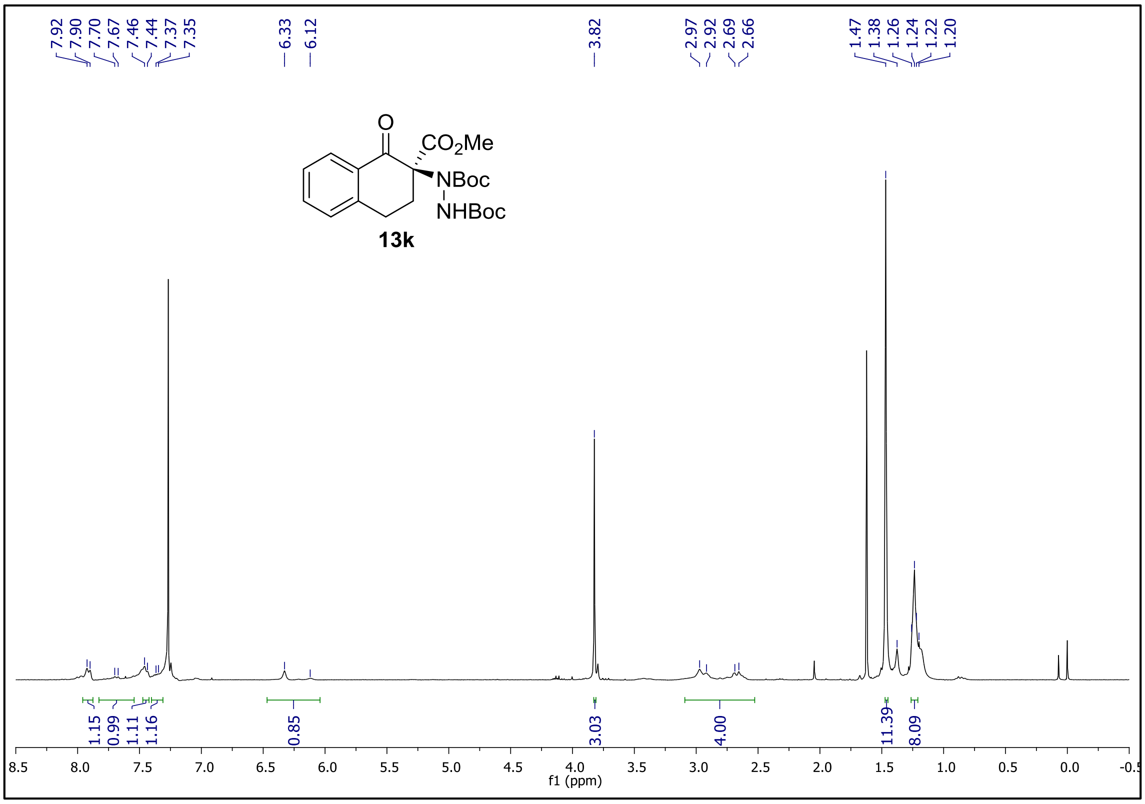
7.92 7.90 7.70 7.67 7.46 7.44 7.37 7.35
6.33 6.12
3.82
2.97 2.92 2.69 2.66
1.47 1.38 1.26 1.24 1.22 1.20
O
CO2Me
NBoc
NHBoc
13k
1.15 0.99 1.11 1.16 0.85 3.03 4.00 11.39 8.09
f1 (ppm)

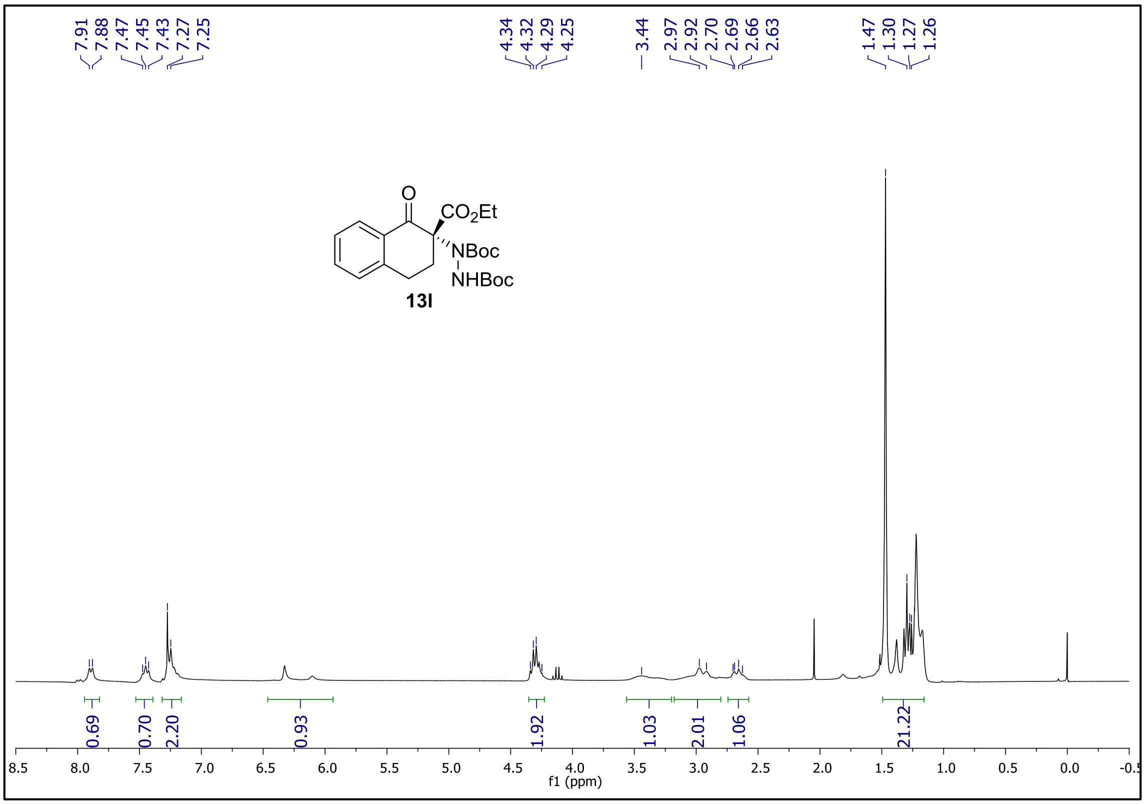
7.91 7.88 7.47 7.45 7.43 7.27 7.25
4.34 4.32 4.29 4.25
3.44
2.97 2.92 2.70 2.69 2.66 2.63
1.47 1.30 1.27 1.26
O
CO2Et
NBoc
NHBoc
13l
0.69 0.70 2.20 0.93 1.92 1.03 2.01 1.06 21.22
f1 (ppm)

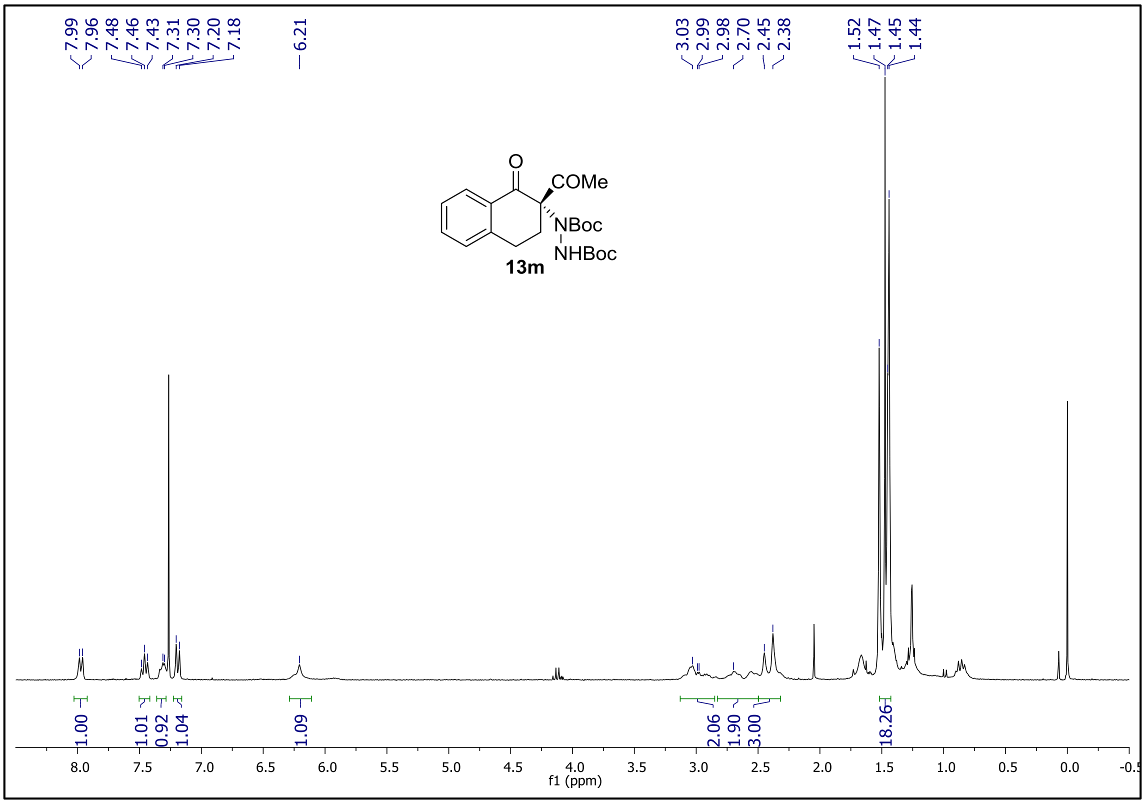
7.99 7.96 7.48 7.46 7.43 7.31 7.30 7.20 7.18
6.21
3.03 2.99 2.98 2.70 2.45 2.38
1.52 1.47 1.45 1.44
COMe
NBoc
NHBoc
13m
1.00 1.01 0.92 1.04 1.09 2.06 1.90 3.00 18.26
f1 (ppm)

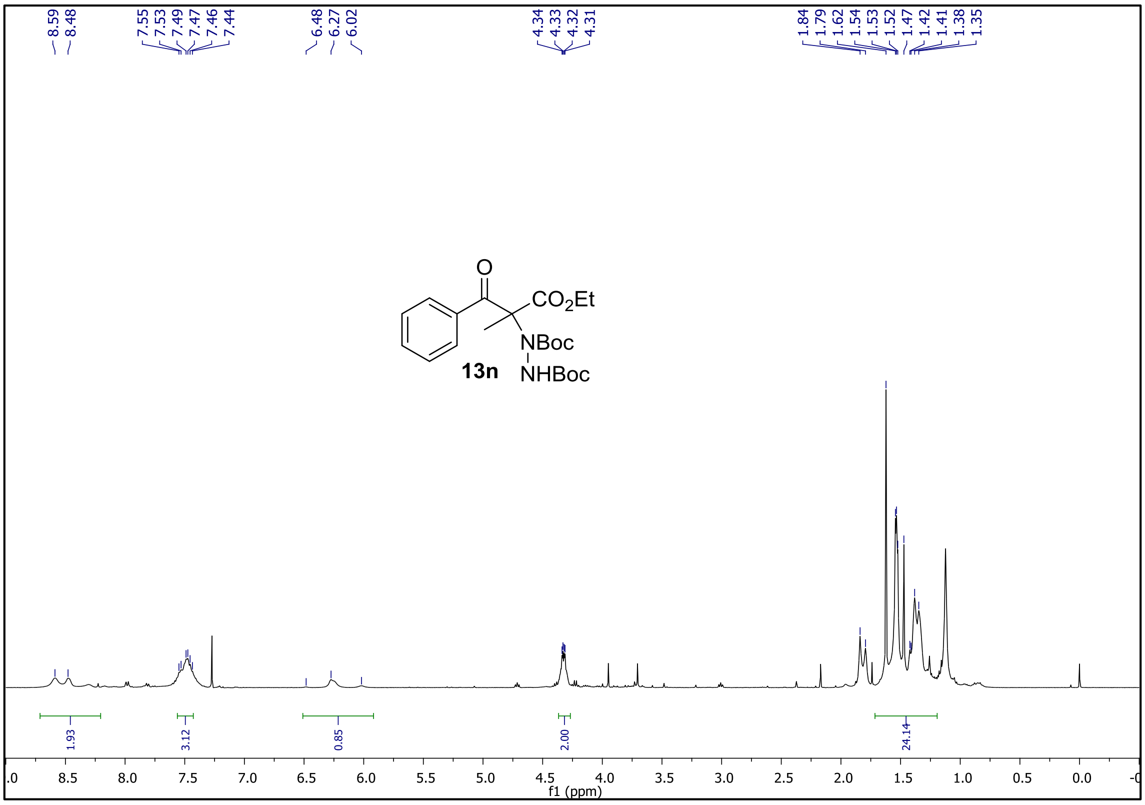
8.59 8.48
7.55 7.53 7.49 7.47 7.46 7.44
6.48 6.27 6.02
4.34 4.33 4.32 4.31
1.84 1.79 1.62 1.54 1.53 1.52 1.47 1.42 1.41 1.38 1.35
CO_2Et
NBoc
13n
NHBoc
1.93 3.12 0.85 2.00 24.14
f1 (ppm)

HPLC Chromatograms of Chiral Amination Products

Only copy of HPLC charts of those enantioenriched products (≥ 20% *ee*) are provided.

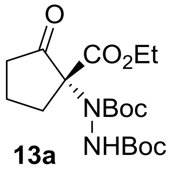

13a

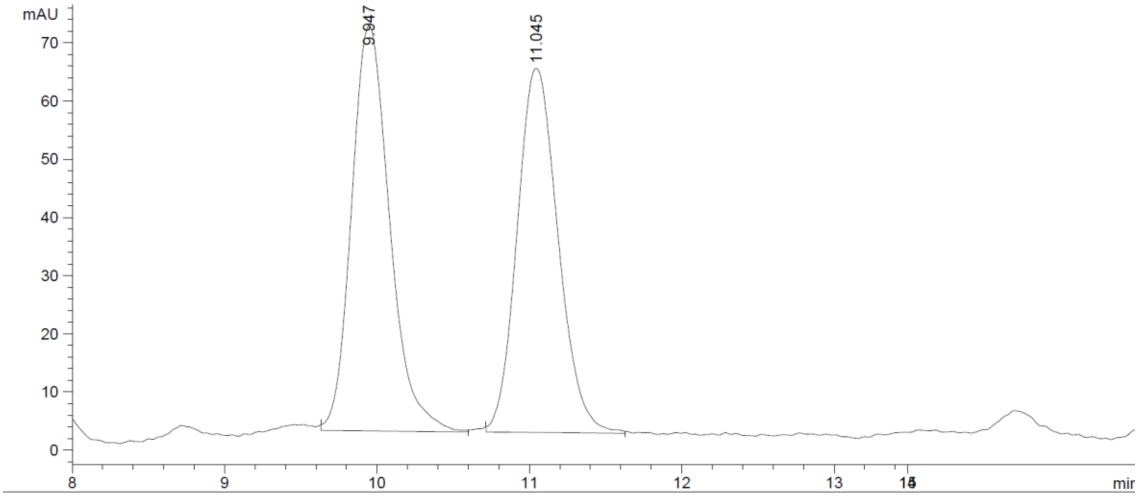

Peak #	RetTime [min]	Type	Width [min]	Area [mAU*s]	Height [mAU]	Area %
1	9.947	BB	0.2676	1215.31445	69.62508	50.9333
2	11.045	BB	0.2902	1170.77722	62.59072	49.0667

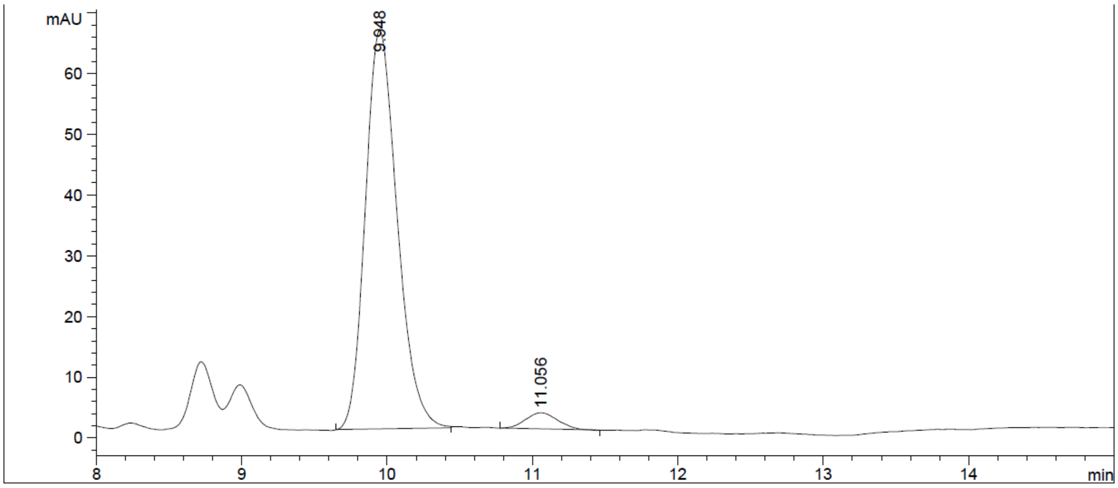

Peak #	RetTime [min]	Type	Width [min]	Area [mAU*s]	Height [mAU]	Area %
1	9.948	PB	0.2363	1002.44684	65.58751	96.0346
2	11.056	PP	0.2359	41.39199	2.68403	3.9654

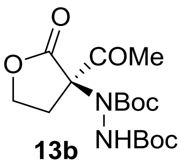

13b

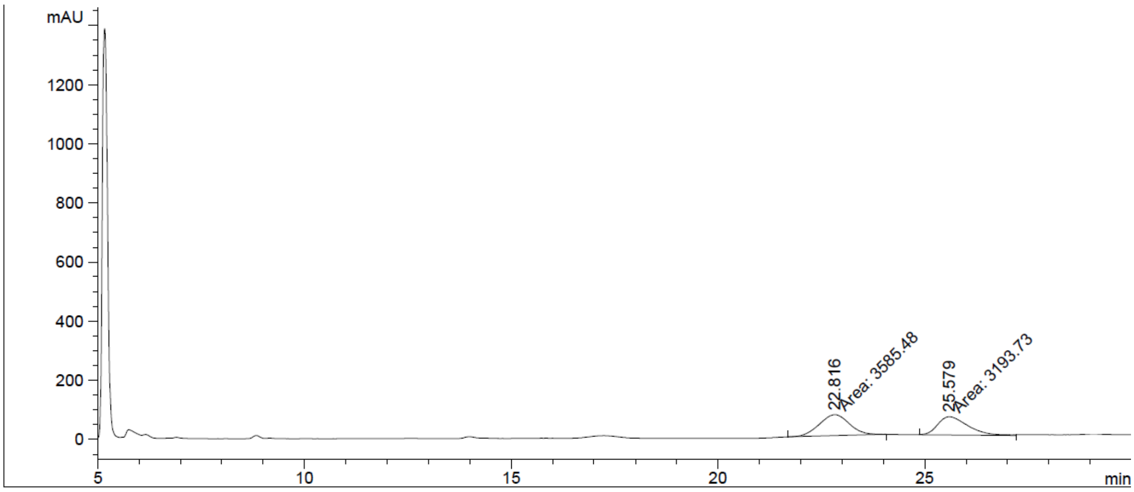

Peak #	RetTime [min]	Type	Width [min]	Area [mAU*s]	Height [mAU]	Area %
1	22.816	MM	0.8439	3585.48389	70.81393	52.8894
2	25.579	MM	0.8556	3193.72705	62.21344	47.1106

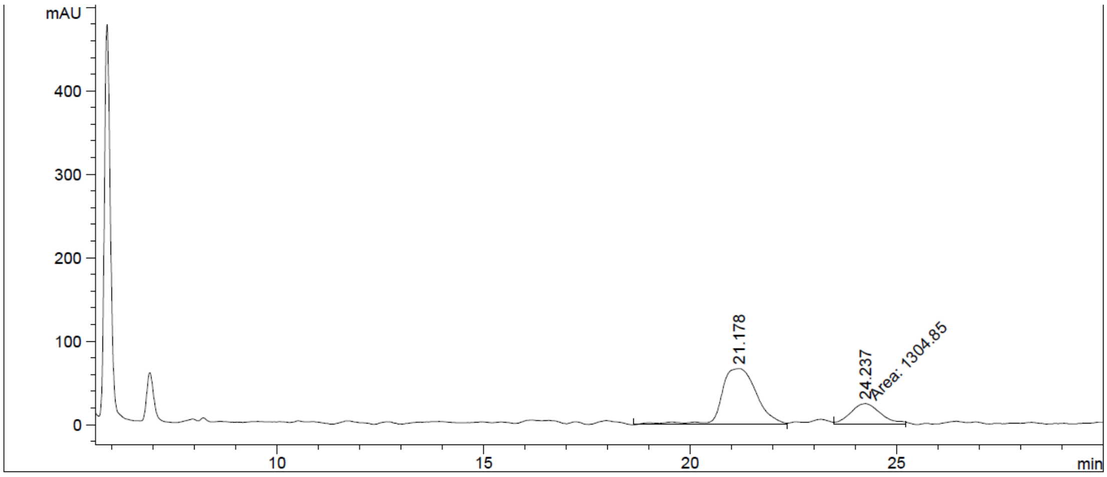

Peak #	RetTime [min]	Type	Width [min]	Area [mAU*s]	Height [mAU]	Area %
1	21.178	VV	0.8528	4010.31909	67.14988	75.4504
2	24.237	MF	0.8654	1304.85059	25.12862	24.5496

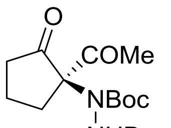

13c

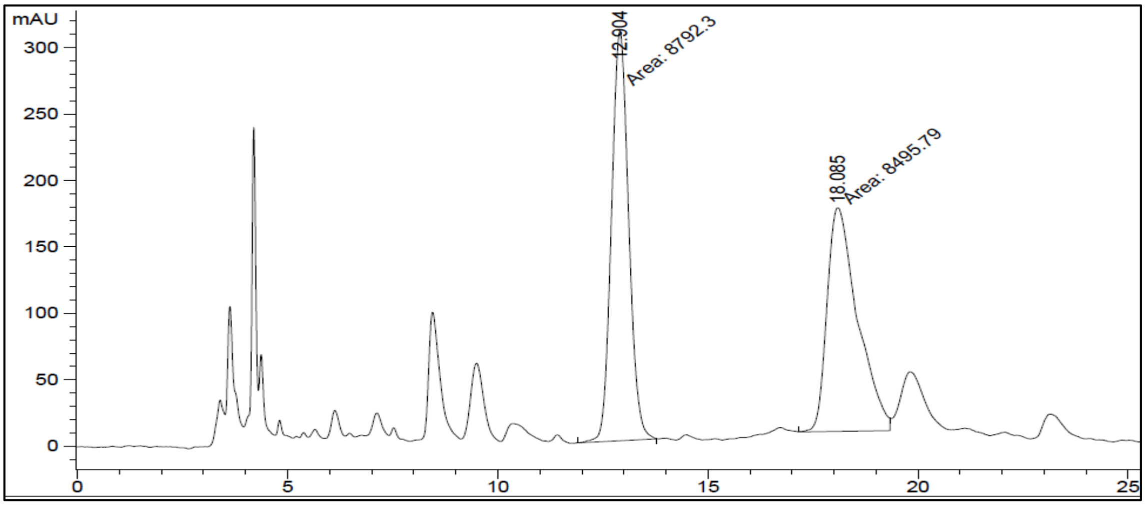

Peak	Ret. Time (min)	Type	Width	Area (mAU*s)	Heigh (mAU)	Area (%)
1	12.904	MM	0.4756	8792.30173	308.11707	50.8576
2	18.085	MF	0.8410	8495.78906	168.36839	49.1424

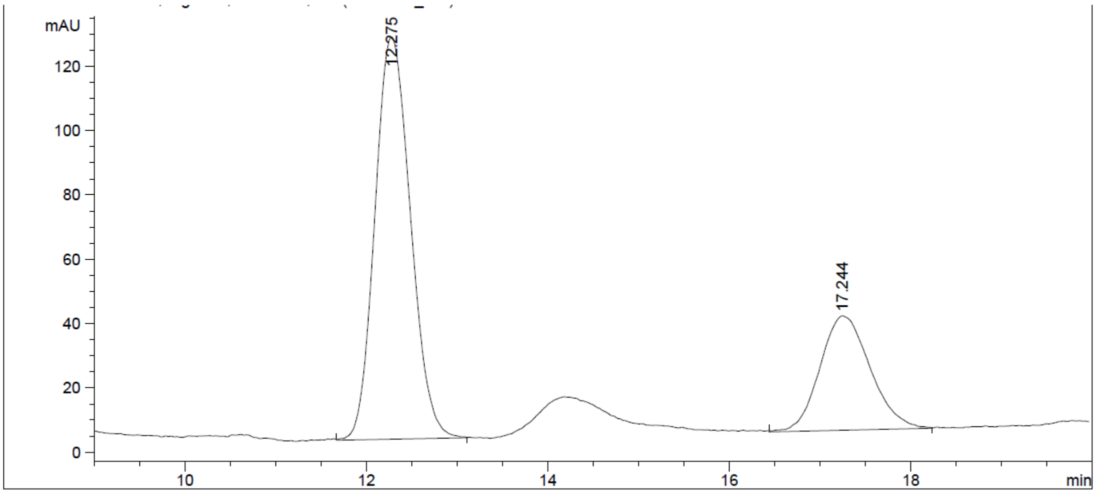

Peak #	RetTime [min]	Type	Width [min]	Area [mAU*s]	Height [mAU]	Area %
1	12.275	VB	0.4244	3415.51733	125.14387	71.3517
2	17.244	BV	0.4829	1371.36121	35.63898	28.6483

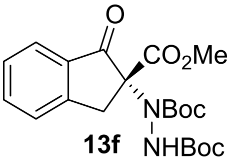

13f

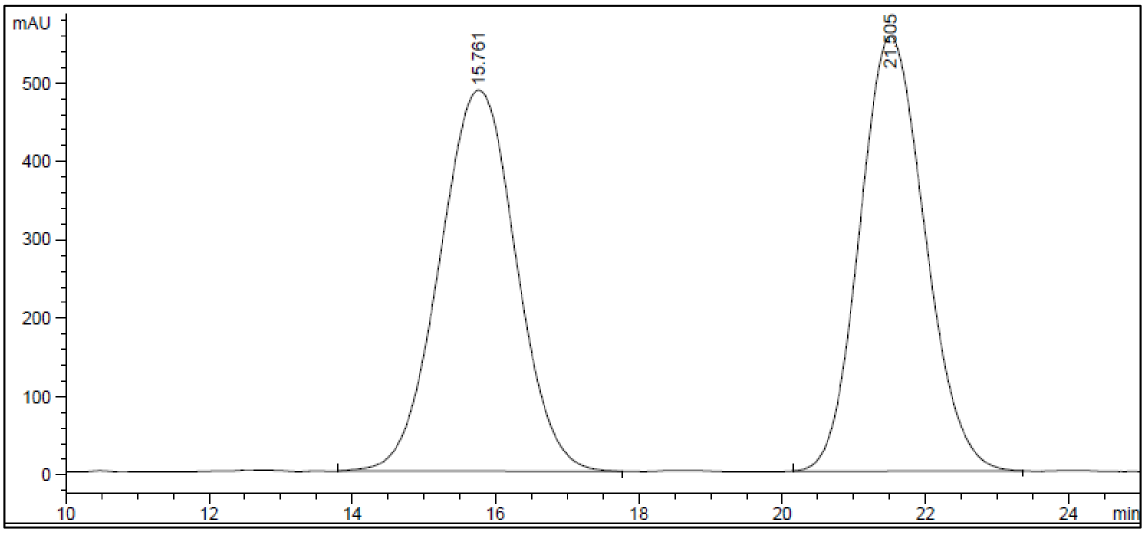

Peak	Ret. Time (min)	Type	Width	Area (mAU*s)	Heigh (mAU)	Area (%)
1	15.761	BB	1.1321	36074.2	485.95621	50.6380
2	21.505	BB	0.9834	35165.2	554.86438	49.3620

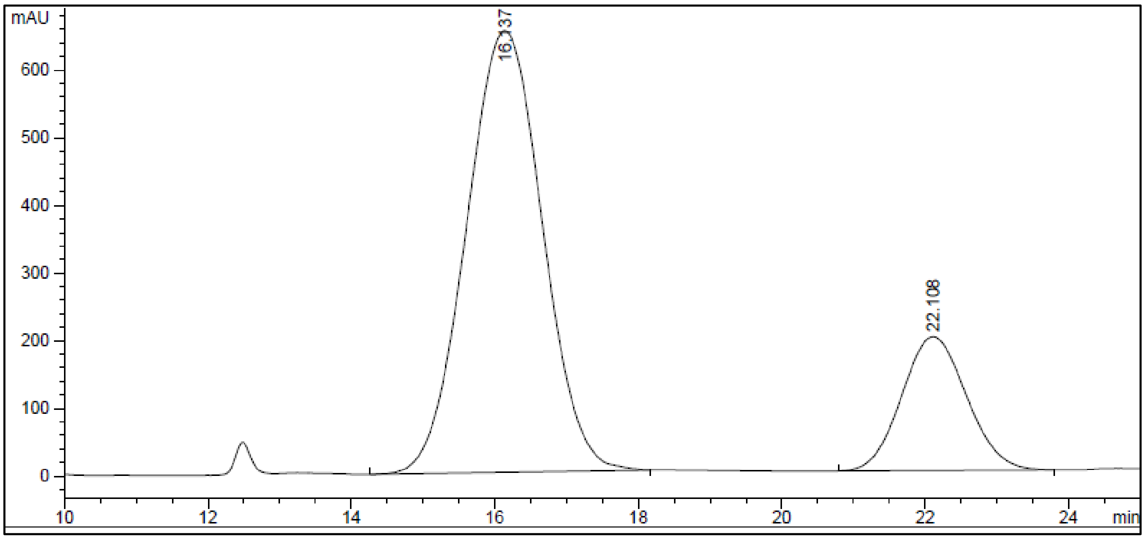

Peak	Ret. Time (min)	Type	Width	Area (mAU*s)	Heigh (mAU)	Area (%)
1	16.137	PB	1.1152	48044.7	652.38928	79.4215
2	22.108	BB	0.9621	12448.6	197.84103	20.5785

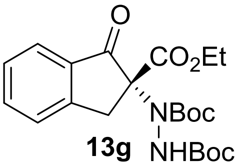

13g

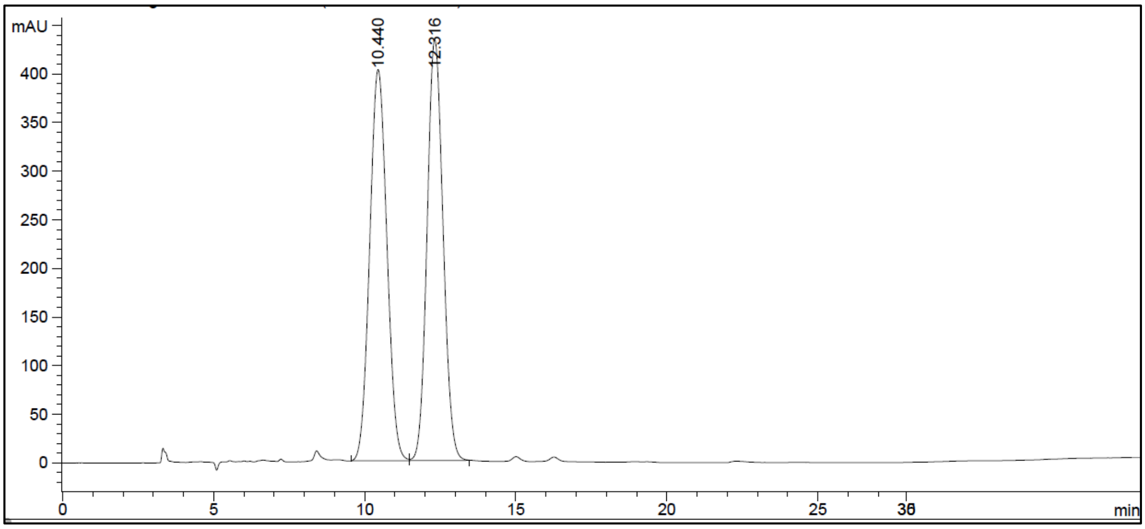

Peak	Ret. Time (min)	Type	Width	Area (mAU*s)	Heigh (mAU)	Area (%)
1	10.440	PV	0.6243	15943.6	402.39029	50.0770
2	12.316	VB	0.5780	15894.5	433.22595	49.9230

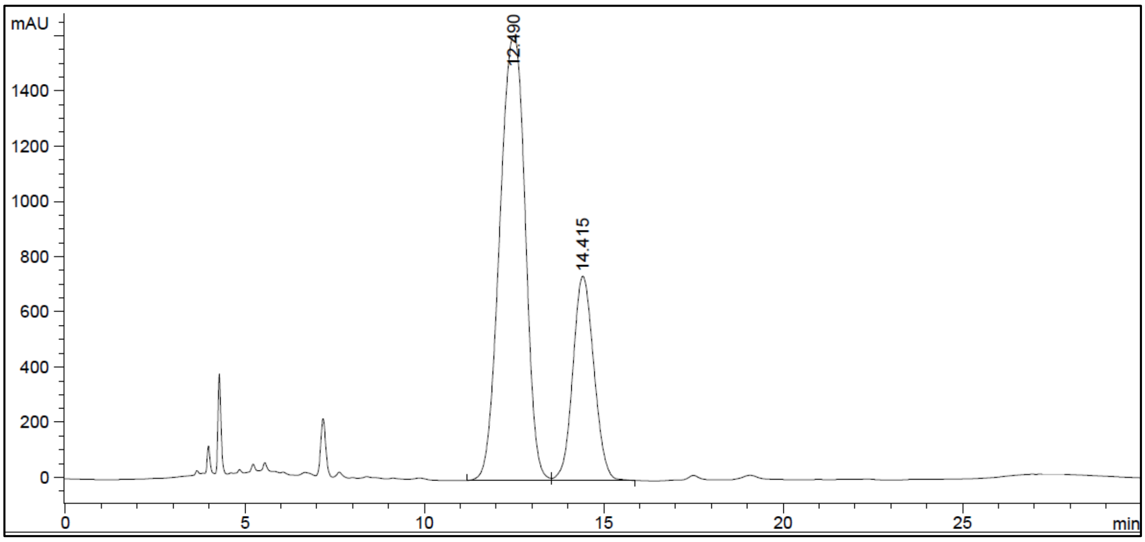

Peak	Ret. Time (min)	Type	Width	Area (mAU*s)	Heigh (mAU)	Area (%)
1	12.490	BV	0.7864	79290.0	1610.40979	72.3872
2	14.415	VB	0.6226	30245.9	740.44135	27.6128

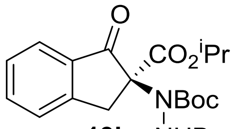

13h

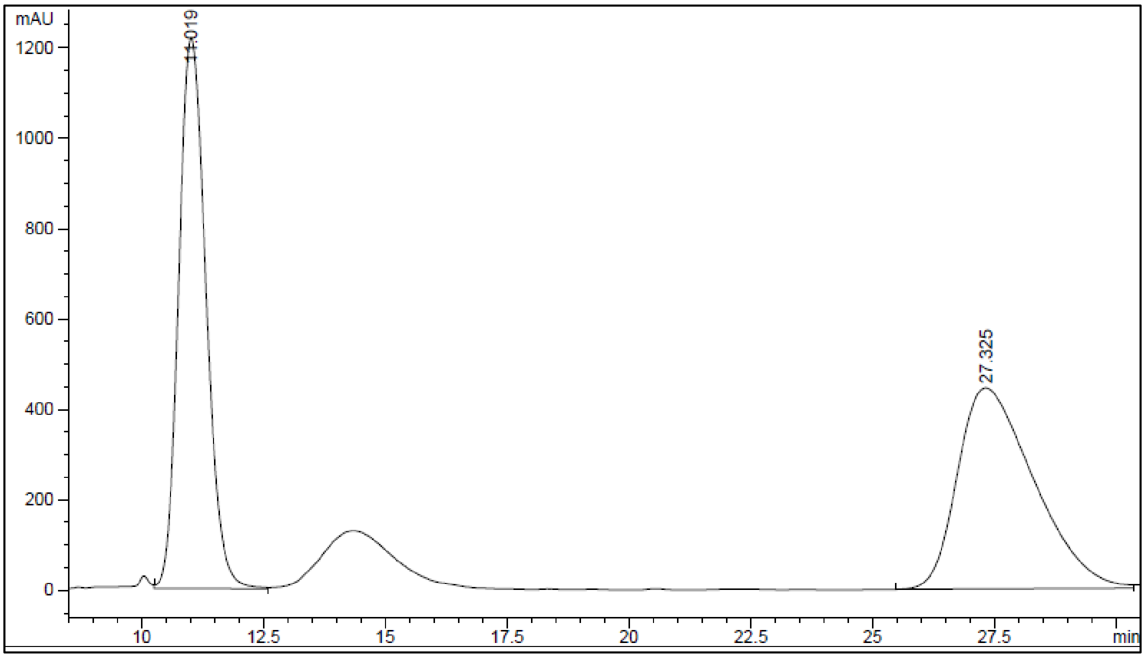

Peak	Ret. Time (min)	Type	Width	Area (mAU*s)	Heigh (mAU)	Area (%)
1	11.019	VB	0.6069	47501.5	1218.22546	49.4937
2	27.325	BB	1.4925	48473.3	444.40399	50.5063

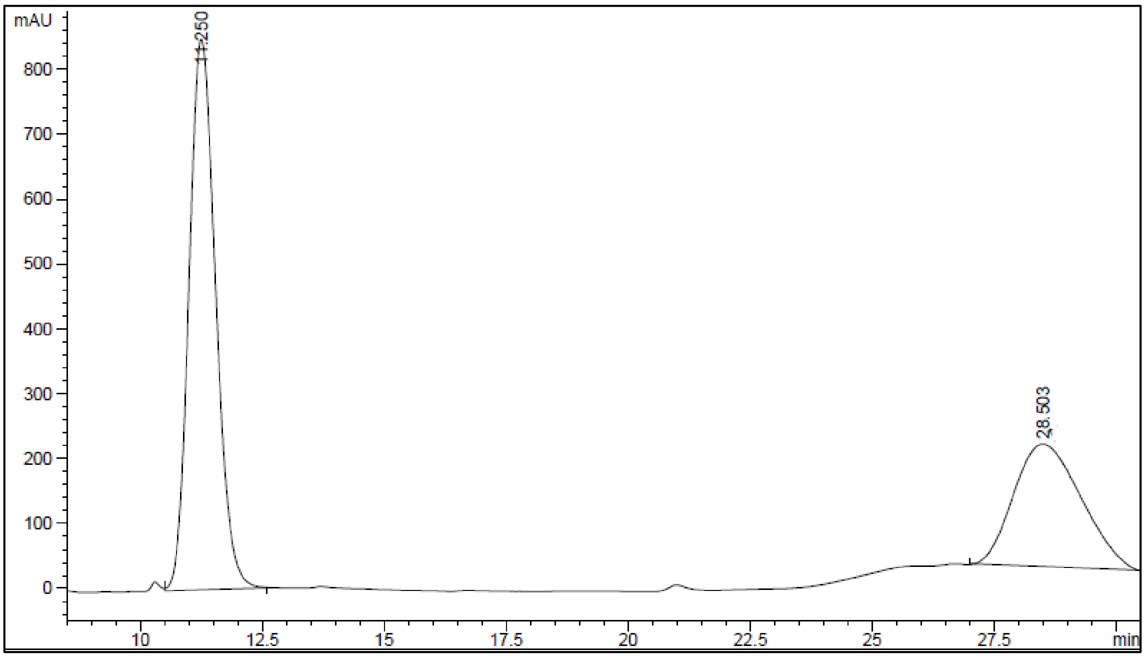

Peak	Ret. Time (min)	Type	Width	Area (mAU*s)	Heigh (mAU)	Area (%)
1	11.250	VB	0.5807	32205.2	848.79523	64.0901
2	28.503	MM	1.5953	18044.7	188.52483	35.9099

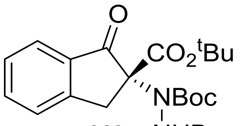

13i

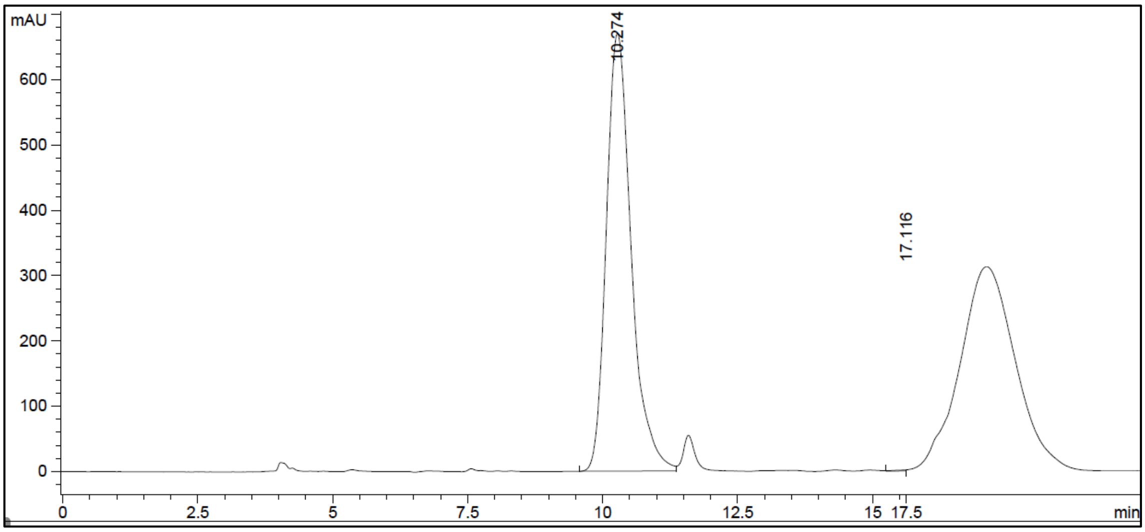

Peak	Ret. Time (min)	Type	Width	Area (mAU*s)	Heigh (mAU)	Area (%)
1	10.274	PV	0.4926	21531.9	670.54132	48.981
2	17.116	VB	1.0154	22427.8	313.21094	51.019

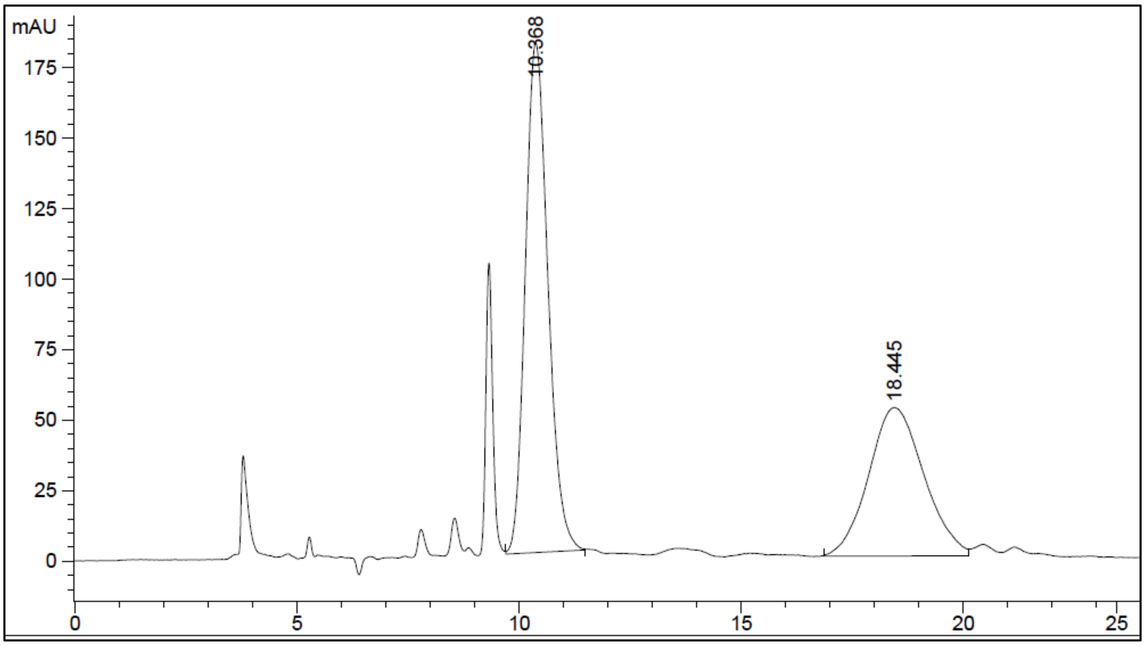

Peak	Ret. Time (min)	Type	Width	Area (mAU*s)	Heigh (mAU)	Area (%)
1	10.368	VB	0.5407	6396.97754	180.82336	58.3856
2	18.445	BV	1.0255	4559.45264	52.69088	41.6144

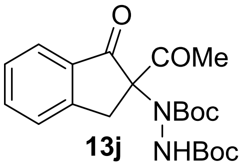

13j

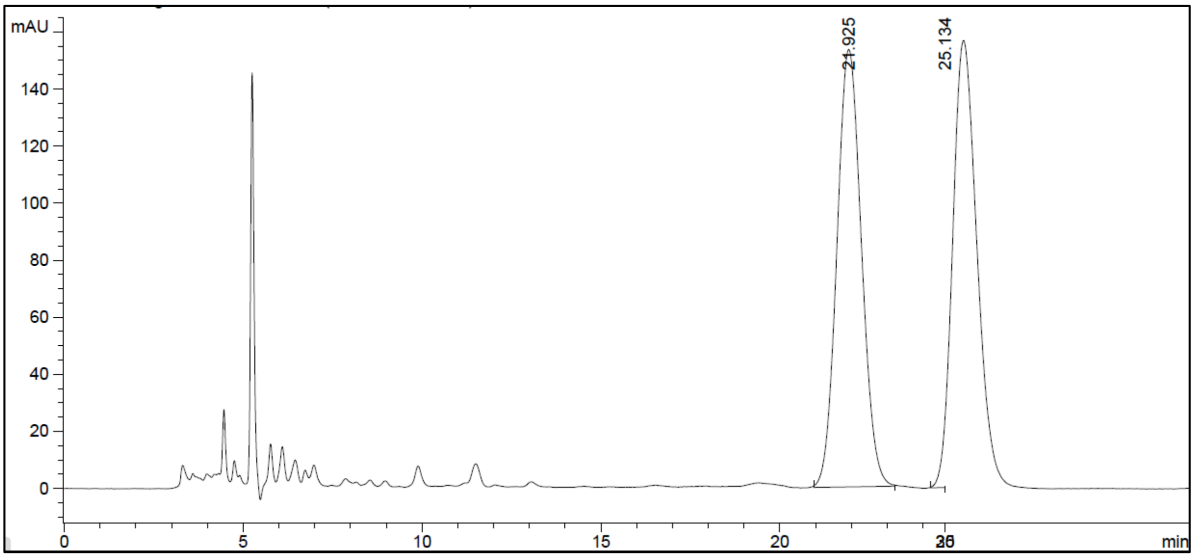

Peak	Ret. Time (min)	Type	Width	Area (mAU*s)	Heigh (mAU)	Area (%)
1	21.925	BB	0.7406	7315.40381	153.34898	49.8468
2	25.134	BB	0.7197	7360.36768	156.77203	50.1532

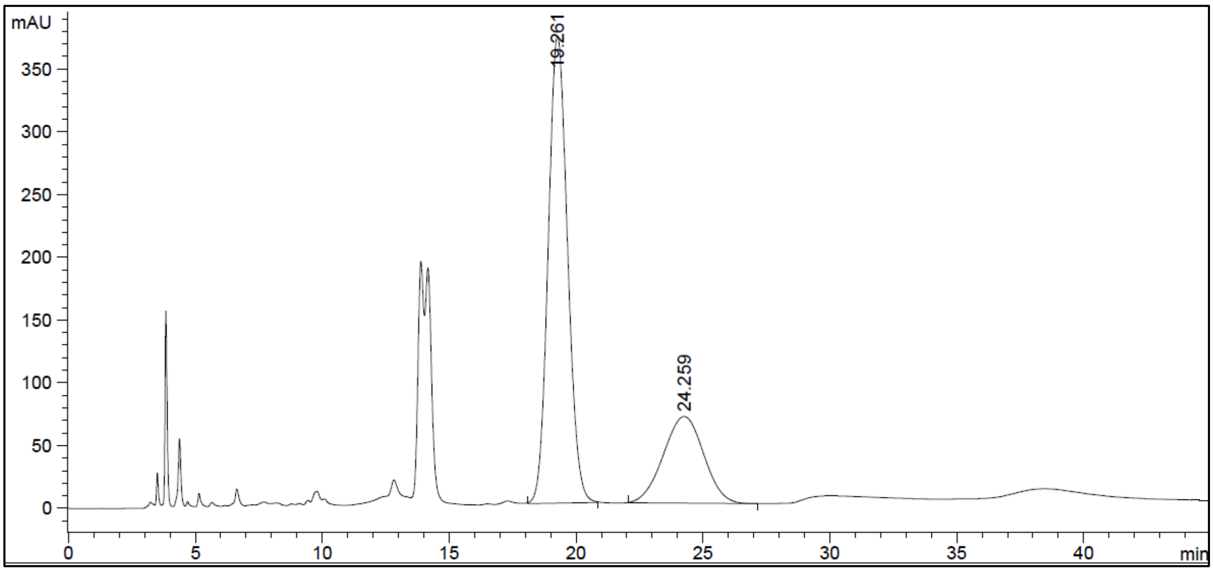

Peak	Ret. Time (min)	Type	Width	Area (mAU*s)	Heigh (mAU)	Area (%)
1	19.261	BB	0.8255	19711.1	372.43018	72.0580
2	24.259	BP	1.4129	7643.38232	69.07708	27.9420

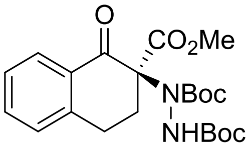

13k

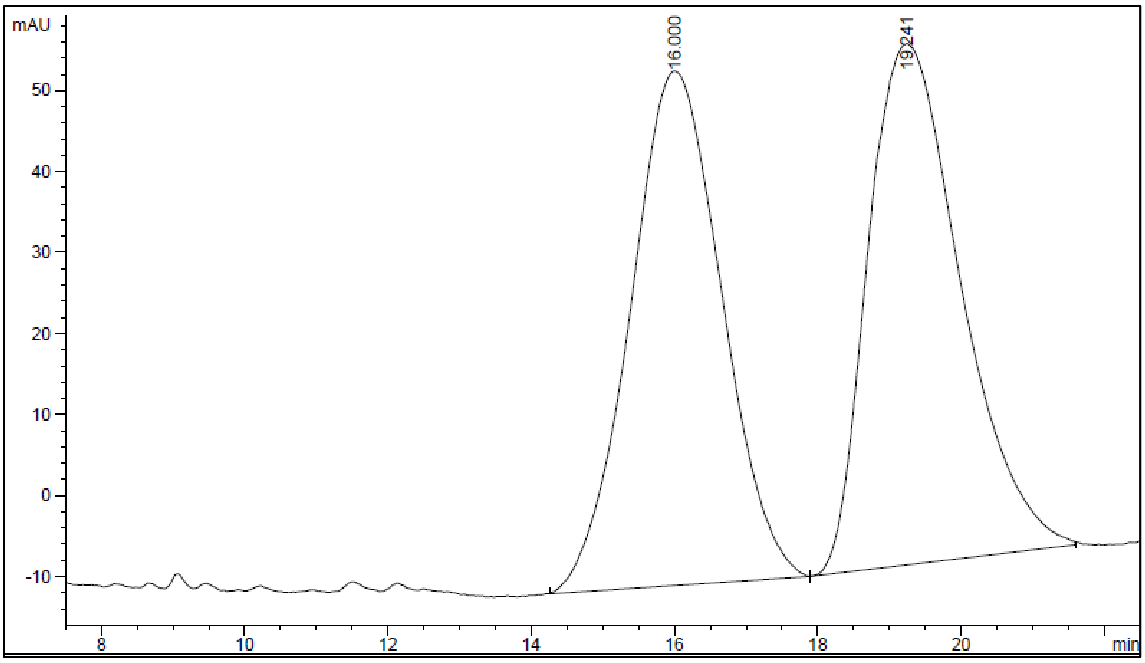

Peak	Ret. Time (min)	Type	Width	Area (mAU*s)	Heigh (mAU)	Area (%)
1	16.000	BP	1.0754	5612.87598	63.48714	49.2383
2	19.241	VB	1.0711	5786.52686	64.35174	50.7617

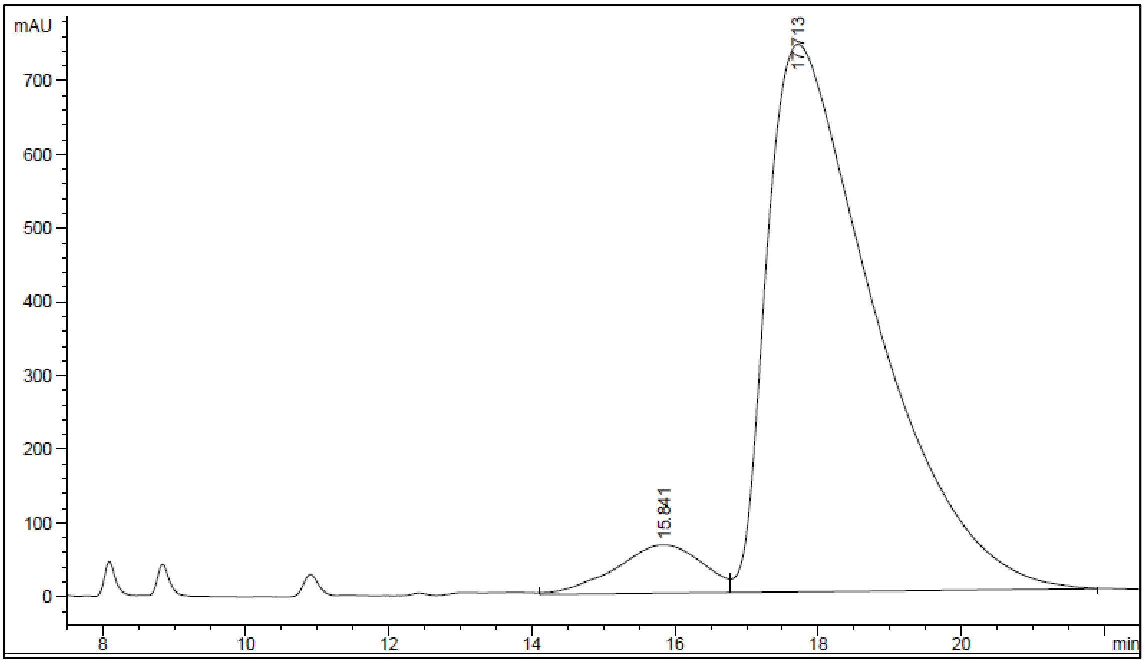

Peak	Ret. Time (min)	Type	Width	Area (mAU*s)	Heigh (mAU)	Area (%)
1	15.841	BV	1.0726	5508.57031	56.67299	6.6035
2	17.713	VB	1.4819	77910.1	742.65242	93.3965

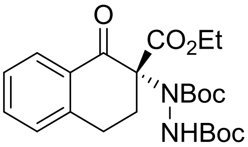

13l

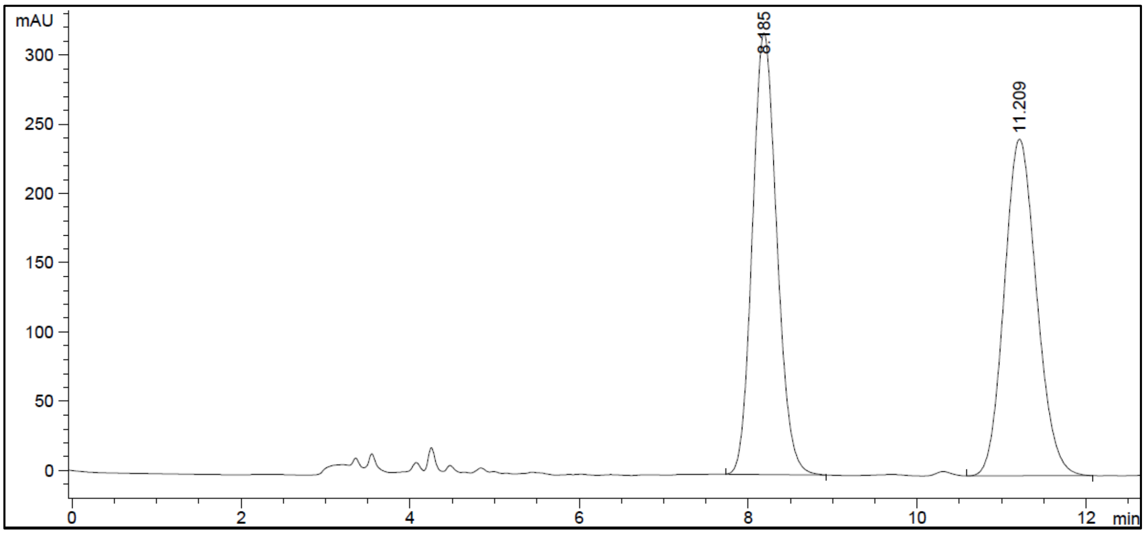

Peak	Ret. Time (min)	Type	Width	Area (mAU*s)	Heigh (mAU)	Area (%)
1	8.185	BB	0.3109	6422.34766	318.93024	49.7649
2	11.209	VB	0.4161	6483.03906	24.11255	50.2351

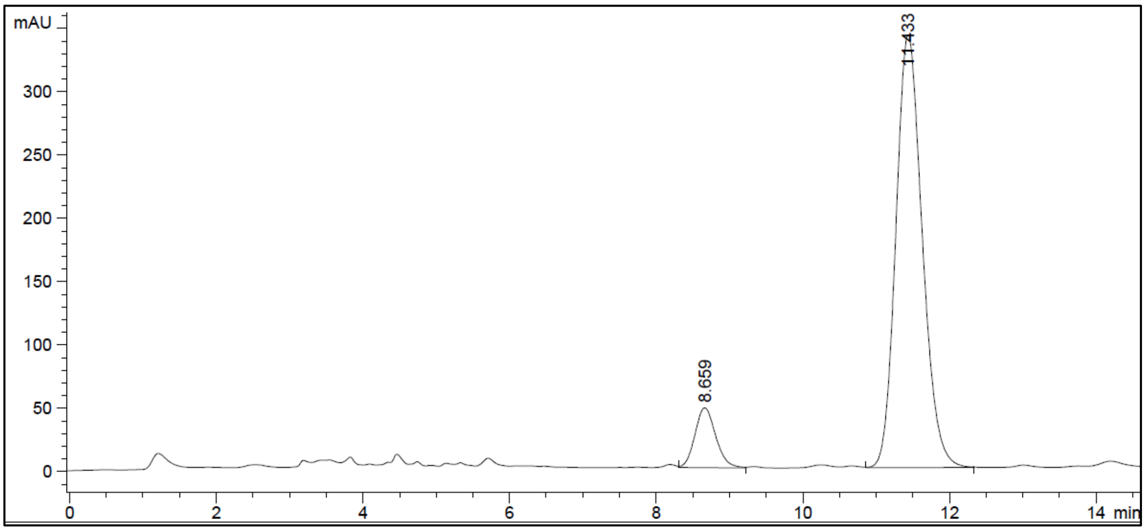

Peak	Ret. Time (min)	Type	Width	Area (mAU*s)	Heigh (mAU)	Area (%)
1	8.659	VB	0.3025	936.74677	47.40308	9.8867
2	11.433	VB	0.3874	8538.04395	341.81696	90.1133

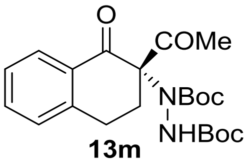

13m

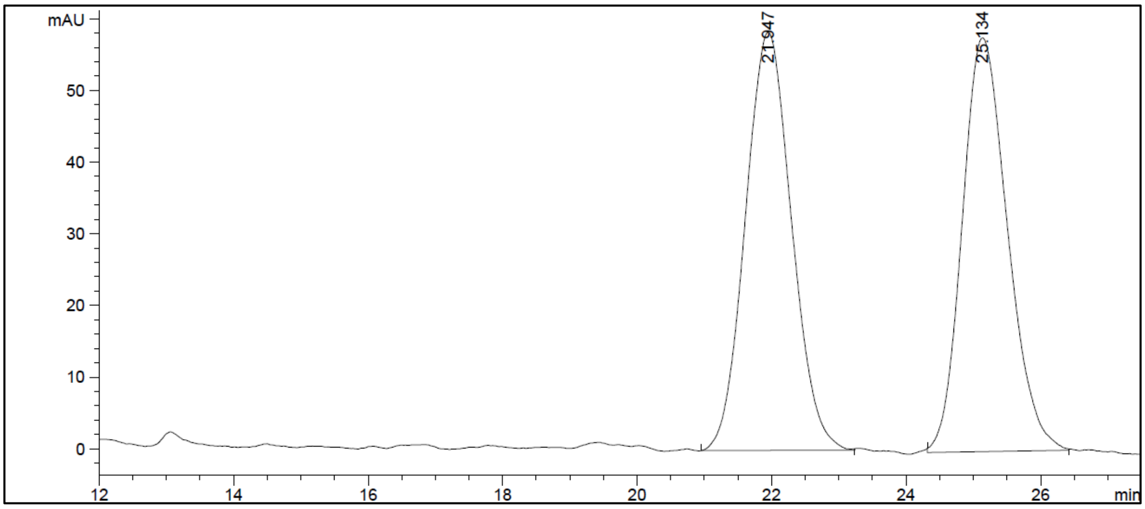

Peak	Ret. Time (min)	Type	Width	Area (mAU*s)	Heigh (mAU)	Area (%)
1	21.947	PB	0.7343	2783.85034	58.38541	50.7383
2	25.134	BB	0.7101	2702.83691	57.74236	49.2617

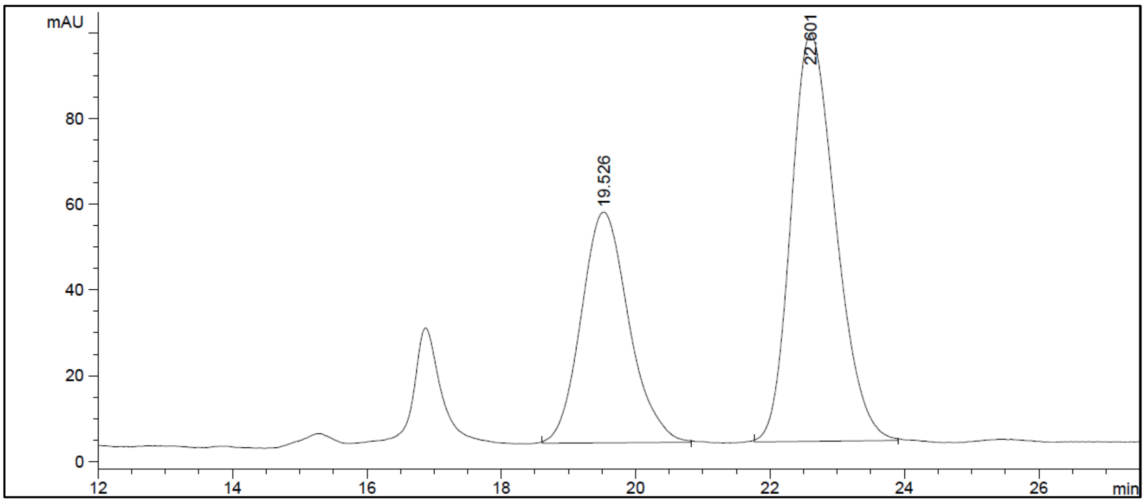

Peak	Ret. Time (min)	Type	Width	Area (mAU*s)	Heigh (mAU)	Area (%)
1	19.526	BB	0.7467	2650.56055	53.80379	37.6096
2	22.601	BB	0.7137	4396.99707	95.05746	62.3904

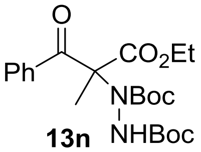

13n

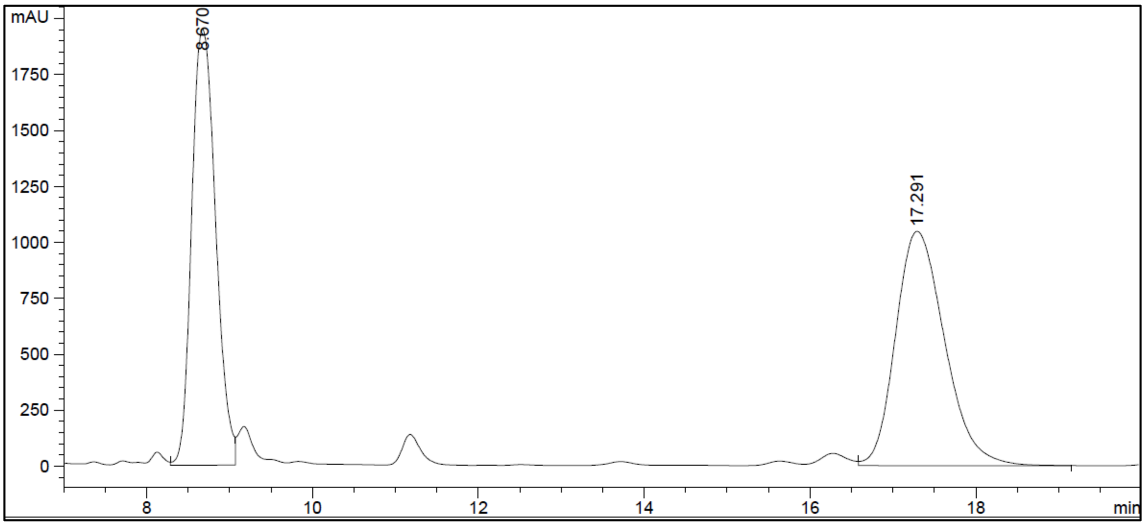

Peak	Ret. Time (min)	Type	Width	Area (mAU*s)	Heigh (mAU)	Area (%)
1	8.670	VV	0.3188	39240.3	1949.99158	47.5930
2	17.291	VB	0.6450	43209.4	1047.99988	52.4070

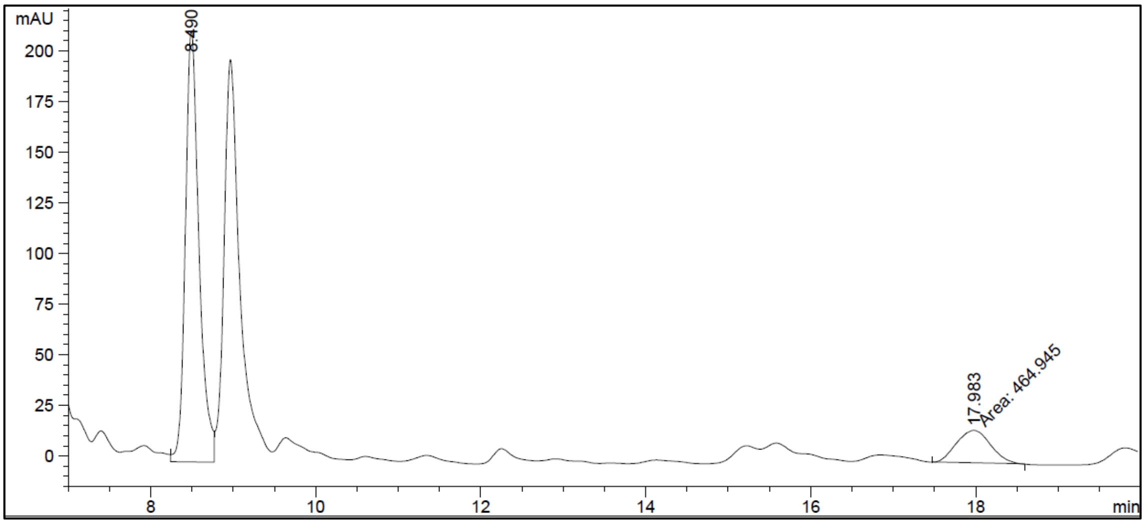

Peak	Ret. Time (min)	Type	Width	Area (mAU*s)	Heigh (mAU)	Area (%)
1	8.490	VV	0.1727	2460.12964	213.02098	84.1049
2	17.983	MM	0.4817	464.94467	16.08597	15.8951
